# Evidence of water on the lunar surface from Chang’E-5 in-situ spectra and returned samples

**DOI:** 10.1038/s41467-022-30807-5

**Published:** 2022-06-14

**Authors:** Jianjun Liu, Bin Liu, Xin Ren, Chunlai Li, Rong Shu, Lin Guo, Songzheng Yu, Qin Zhou, Dawei Liu, Xingguo Zeng, Xingye Gao, Guangliang Zhang, Wei Yan, Hongbo Zhang, Lihui Jia, Shifeng Jin, Chunhua Xu, Xiangjin Deng, Jianfeng Xie, Jianfeng Yang, Changning Huang, Wei Zuo, Yan Su, Weibin Wen, Ziyuan Ouyang

**Affiliations:** 1 Key Laboratory of Lunar and Deep Space Exploration, National Astronomical Observatories, Chinese Academy of Sciences, Beijing, 100101 China; 2 University of Chinese Academy of Sciences, Beijing, 100049 China; 3 Key Laboratory of Space Active Opto-electronics Technology, Shanghai Institute of Technical Physics, Chinese Academy of Sciences, Shanghai, 200083 China; 4 State Key Laboratory of Lithospheric Evolution, Institute of Geology and Geophysics, Chinese Academy of Sciences, Beijing, 100029 China; 5 Beijing National Laboratory for Condensed Matter Physics, Institute of Physics, Chinese Academy of Sciences, Beijing, 100190 China; 6 Beijing Institute of Spacecraft System Engineering, Beijing, 100094 China; 7 Beijing Aerospace Control Center, Beijing, 100094 China; 8 Xi’an Institute of Optics and Precision Mechanics, Chinese Academy of Sciences, Xi’an, 710119 China; 9 Beijing Institute of Space Mechanics Electricity, China Academy of Space Technology, Beijing, 100076 China; 10 Institute of Geochemistry, Chinese Academy of Sciences, Guiyang, 550081 China

**Keywords:** Planetary science, Planetary science

## Abstract

The distribution range, time-varying characteristics, and sources of lunar water are still controversial. Here we show the Chang’E-5 in-situ spectral observations of lunar water under Earth’s magnetosphere shielding and relatively high temperatures. Our results show the hydroxyl contents of lunar soils in Chang’E-5 landing site are with a mean value of 28.5 ppm, which is on the weak end of lunar hydration features. This is consistent with the predictions from remote sensing and ground-based telescopic data. Laboratory analysis of the Chang’E-5 returned samples also provide critical clues to the possible sources of these hydroxyl contents. Much less agglutinate glass contents suggest a weak contribution of solar wind implantation. Besides, the apatite present in the samples can provide hydroxyl contents in the range of 0 to 179 ± 13 ppm, which shows compelling evidence that, the hydroxyl-containing apatite may be an important source for the excess hydroxyl observed at this young mare region.

## Abstract (web summary)

Laboratory analysis of returned Chang’E-5 samples from the lunar surface show their hydroxyl contents to be on the weak end of lunar hydration features.

## Introduction

A wide range of evidences indicates the presence of water (H_2_O/OH) on the lunar surface from a diversity of potential sources. Determining the spatial distribution and temporal characteristics of water and its sources is key to understanding lunar magma ocean evolution, mantle volatile content, bombardment history, and the interactions between the solar wind and the lunar surface. Detection of water on the Moon is considered to be one of the most important discoveries in planetary science and a crucial milestone for lunar scientific research^1^. Lunar water is not only an important key to the formation and evolution of the Moon itself, but also provides significant information about the evolution of the solar system^2^. In addition, its presence is expected to provide support for future human lunar in situ resources.

It was initially believed that the Moon was extremely dry^3–6^, However, this view has changed since the neutron spectrometers detect the global presence of H within the first meter below the surface, especially enrichment of H at the poles and the permanently shaded regions^7,8^. Subsequently, the presence of lunar water has been found by many additional spectrometers including the Moon Mineralogy Mapper (M^3^) instrument on the Chandrayaan-1 mission, high-resolution instrument infrared spectrometer (HRIIR) on the Deep Impact/EPOXI mission, the visual and infrared mapping spectrometer (VIMS) on the Cassini spacecraft, and the OSIRIS-REx Visible and InfraRed Spectrometer (OVIRS) on the OSIRIS-REx mission^9–16^. NIR spectral analysis of the LCROSS ejecta plume also discovered the water in the permanently shaded regions^17^. Direct evidence of water on the lunar surface was also confirmed through the analysis of the Apollo samples, which shows that water can exist in the pristine volcanic glass beads, olivine-hosted melt inclusions within glasses, apatites, and pristine plagioclase in lunar ferroan anorthosite^18–21^.

Many different sources have been proposed for the water detected on the Moon, including indigenous water^15,20,21^, solar wind implantation^9–12,22,23^, and meteorite or comet impacts^19^. Recently, some researchers have also suggested that lunar water may originate from “Earth wind”^24^, or the release of exospheric dust impacts^25^, further complicating an understanding of its origin.

Most hyperspectral remote sensing results support the theory of solar wind implantation^9–14^. The H ions in the solar wind bombard the lunar surface at high speed (300–800 m/s), and then are injected into the surface to form OH or H_2_O with free O bonds created probably by solar wind particle sputtering and implantation, micrometeorite vaporization, solar UV radiation or cosmic and galactic ray spallation and implantation^26^. The content of water (mainly OH) formed by this interaction may exhibit a diurnal effect, which suggests a dynamic surface hydroxylation^27^, and the distribution of water increase with latitude, reaching its highest values in polar regions^10,12^. In addition, minerals (such as apatite) containing indigenous water are considered to be present in small quantities and unevenly distributed on the lunar surface, and thus may not be readily detected by remote sensing spectroscopy^26^. However, magmatic indigenous water was detected in the central peak of Bullialdus Crater^28^. Milliken and Li also found widespread indigenous water in lunar pyroclastic deposits at low lunar latitudes (<30°) using M^3^ hyperspectral data^15^. These lines of evidence prove that the spectral signals of indigenous water could be detected by hyperspectral remote sensing. In contrast, the recent results of Bandfield et al. still maintained that the solar wind is the main source of lunar water based on a new thermal-physical correction model^22^, and the hydration can be presented at all latitudes, local times, and surface types, without significant diurnal migration. Thus, Bandfield et al. concluded that the surface water can exist stably in the lattice defects of minerals, and an indigenous source of water is not necessary^22^.

The sources and distributions of water on the Moon is still an open question with no consensus. Unlike the previous studies most based on the orbital remote sensing spectral measurements, Chang’E-5, which successfully landed in northeast Oceanus Procellarum at 15:11 on December 1, 2020 (UTC time, landing site: 51.9160°W, 43.0581°N, elevation: −2550 m), provided a unique opportunity to in-situ investigate the water on the Moon. Here we show new evidence of water on the lunar surface based on Chang’E-5 in situ spectra and laboratory results of returned samples. Our results show the hydroxyl contents of lunar soils in Chang’E-5 landing site are with a mean value of 28.5 ppm, which is on the weak end of lunar hydration features. The hydroxyl-containing apatite may be an important source for the excess hydroxyl observed at this young mare region.

## Results and discussion

### Evidence of water from LMS in situ spectra

**Fig. 1:**
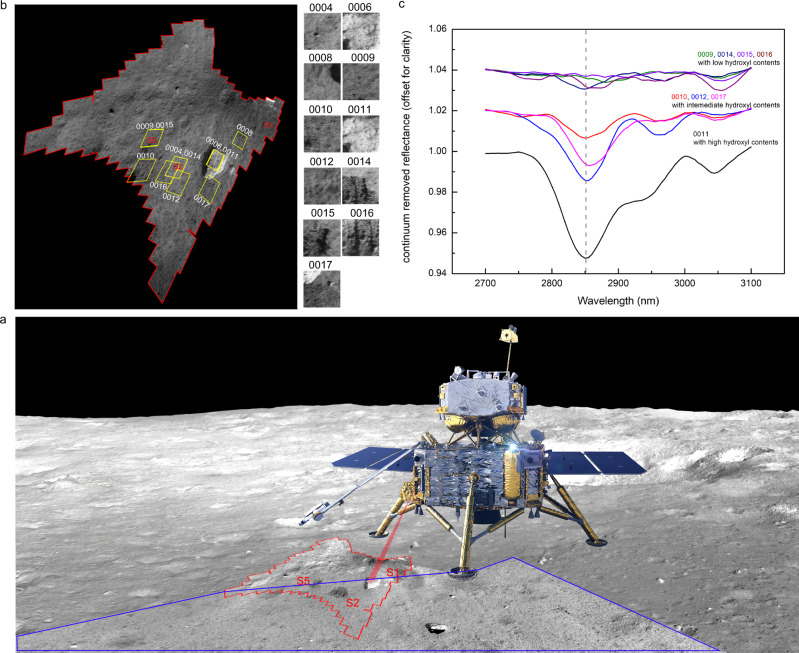
Schematic diagram of the lunar mineralogical spectrometer (LMS) spectral data collection on the lunar surface and the 2700–3100 nm absorption features of Chang’E-5 in situ spectra. **a** The area marked by the red line is the full view of LMS, S1, S2, S5 are the scoop sampling points, the blue box identifies the imaging area of the panoramic camera and the base image is from landing camera. **b** detailed positions and images of the LMS 11 Full-Bands Observation (FBO) detections. LMS operated twice at the S2 sampling point (detection number: 0004 and 0014), S5 sampling point (detection number: 0009 and 0015) and the large rock (detection number: 0006 and 0011) in the field of view; the base map is composed of 180 images obtained by the LMS Full-View Scanning and Multispectral Observation (FVSMO) detections with a center wavelength of 750 nm (More details see “Methods”). **c** the 2700–3100 nm continuum removed absorption features of the 8 LMS FBO hyper-spectra (more details see “Methods”). The gray dashed line shows the absorption positions near 2.85 μm.

Chang’E-5 landing site is located at one of the youngest extrusive lava flow units on the Moon^29,30^, and thus any apatite in the mesostasis of these basalts is an ideal target to assess for evidence of indigenous water^26,31^. The Chang’E-5 lander was equipped with the lunar mineralogical spectrometer (LMS), the spectral range of which covered from 0.48 to 3.2 μm (Supplementary Table 1), meaning that it could effectively characterize the absorption features of OH/H_2_O near 3 μm. During the period of scoop sample collection, the LMS acquired 11 hyperspectral data of a rock and different lunar soil targets before and after scoop sampling (Fig. 1a, b), but only 8 of them were employed in this work (Fig. 1c, more details see “Methods”).

The LMS data have been processed by radiometric correction, thermal correction (three methods including Clark et al., Li et al., and Groussin et al. were used separately)^32–34^, reflectance calculation, and photometric correction (see “Methods”). The continuum-removed reflectance spectra in the range of 2700–3100 nm for the 8 representative targets were shown in Fig. 1c, which exhibit absorption features at 2.85 μm, 2.95 μm and 3.05 μm (see “Methods”). The sources about the 2.95 μm and 3.05 μm absorptions are complex and may be related to the absorptions of H_2_O^35^ (see “Methods”).

The most pronounced hydration features at 2.85 μm mainly caused by hydroxyl (OH) were the focus of this work. As shown in Fig. 1c, the LMS in situ spectra exhibit varying degrees of absorption at 2.85 μm. Specially, the absorption feature of the rock (named “ShiGanDang”, detection number: 0011) is much more significant than those of lunar soils. Here we estimated the hydroxyl contents of these 8 targets (see “Methods”, Table 1). The rock spectrum 0011 was estimated to have the highest hydroxyl content, and the relatively high hydroxyl content exhibited by the spectrum 0017 may be due to the fact that part of the rock can be seen in the field of view (Fig. 1b). However, the overall hydroxyl contents of soil spectra except 0012 are very low, with mean value of only 28.5 ppm.

**Table 1:** The characteristics of the lunar mineralogical spectrometer (LMS) detections and estimated hydroxyl contents.

Detection number	Spectral obtaining time (lunar local time)	Derived temperatures on the lunar surface (K) using the model by Clark et al.^32^	Calculated hydroxyl contents (ppm) Using the model by Clark et al.^32^
0009	10:07	344	11
0010	10:08	335	26
0011	10:08	348	152
0012	10:10	341	110
0014	10:20	344	15
0015	10:24	347	2
0016	10:32	347	7
0017	10:33	357	82

### Evidence of hydroxyl from Chang’E-5 returned lunar samples

What is the possible source of hydroxyl found at the Chang’E-5 landing site? Do the returned lunar samples contain hydroxyl or hydroxyl-bearing minerals? We addressed these questions using (1) the engineering qualification module of the LMS instrument (LMS-EQM, parameters, and status are consistent with the LMS on the lunar surface), (2) the scanning electron microscopy (SEM), (3) the energy-dispersive spectrometer (EDS), (4) the X-ray diffraction (XRD), (5) Raman spectrometer, and (6) the electron-probe micro-analysis (EPMA) to further assess the presence and state of hydroxyl or hydroxyl-bearing minerals in the samples returned by Chang’E-5.

Both Chang’E-5 lunar soil powder and rock fragments were randomly selected for laboratory analysis (see “Methods”). Under ambient laboratory conditions, the LMS-EQM was first used to obtain full spectral data for the three samples of lunar soil powder (see “Methods”). As expected, the absorption characteristics near 2.85 μm also existed in the laboratory spectra (Supplementary Fig. 9), indicating that hydroxyl should also present in the Chang’E-5 returned samples, while the broadened features near 3 μm may be attributed to the molecular water in the optical path.

**Fig. 2:**
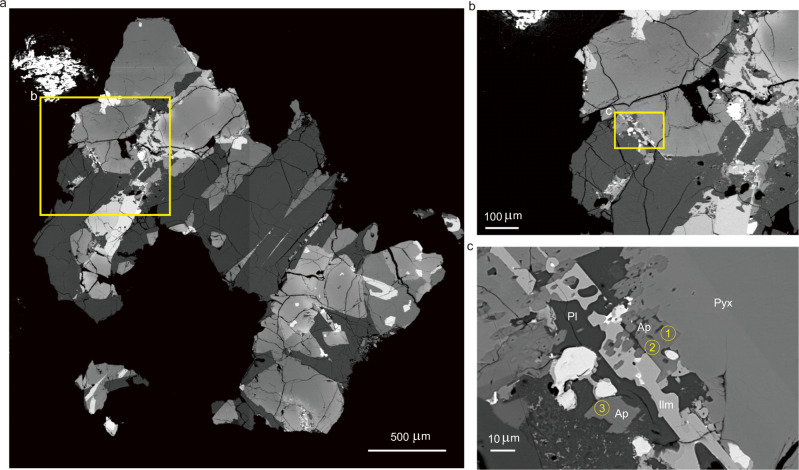
Back-Scattered Electron (BSE) images of representative apatite grains in a basalt clast (sample: CE5C0000YJYX042GP). The basaltic clast is mainly composed of pyroxene, plagioclase, and olivine, but a considerable quantity of apatite and ilmenite are also found. Panel **b** is a zoomed-in image of (**a**), and panel **c** is a zoomed-in image of (**b**), the positions of Raman and EPMA measurements are marked with yellow circled numbers in panel **c**. Pyx-pyroxene; Pl-plagioclase; Ap-apatite; Ilm-Ilmenite.

In order to further assess the possible existence and source of hydroxyl in the lunar samples, the mineral composition of three rock fragments was analyzed by SEM and EDS (see “Methods”). As shown in the back scattering electron (BSE) images of Fig. 2, there is a considerable quantity of apatite grains in addition to the major minerals of pyroxene, plagioclase, olivine, and ilmenite grains.

**Fig. 3:**
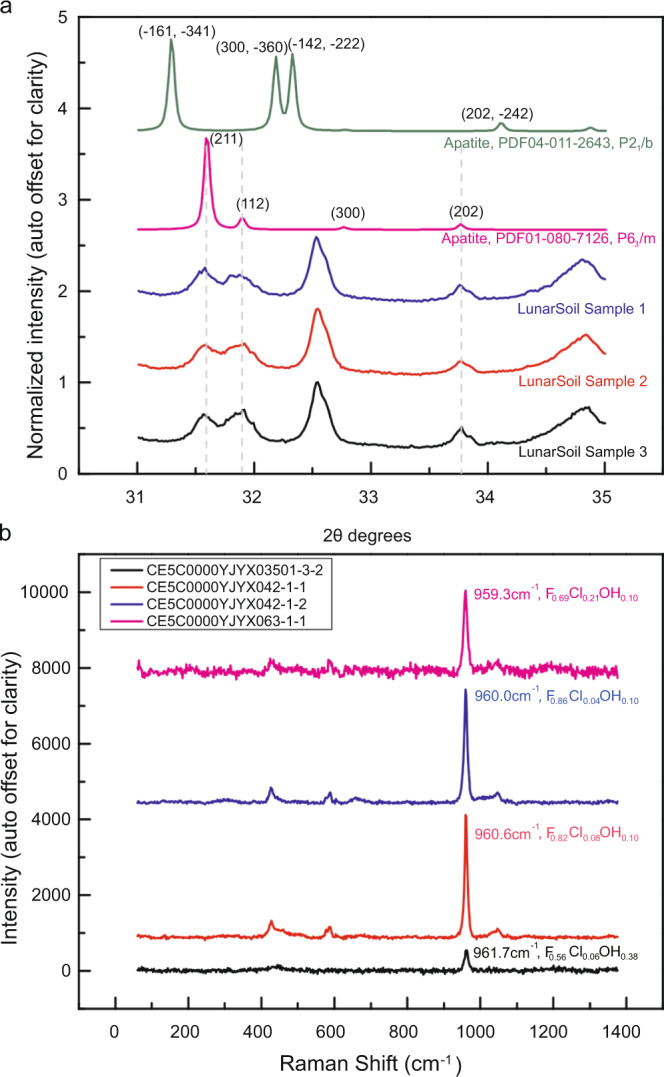
The X-ray diffraction (XRD) measurements of Chang’E-5 lunar soil samples and Raman spectra analysis of Chang’E-5 apatite grains in the polished sections. **a** Identification of apatite in lunar soil samples by matching XRD features with the mineral powder diffraction files (PDF) of the International Center for Diffraction Data (ICDD). There are two kinds of crystal structures in apatite: P6_3_/m and P2_1_/b, and the crystal structure of apatite identified in the lunar soils is P6_3_/m. The gray dashed lines show the diffraction peaks of lunar soils matching result with the standard apatite mineral features of the three crystal planes (211), (112), and (202), and the diffraction pattern of the standard apatite mineral (PDF01-080-7126) has been (h00) preferred orientation corrected. **b** The Raman spectra of different apatite grains in the polished sections. The apatite formulas shown in the figure is derived from the corresponding electron-probe micro-analysis (EPMA) results. We infer that the Raman peak shifts toward 962 cm^−1^ as the proportion of OH in the apatite grains increased. Part of the Raman measurement positions are marked in Fig. 2c.

The mineral phases and abundances of the three lunar soil samples were also determined by multiple analytical methods, including SEM and EDS, XRD and the Rietveld full-pattern fitting method (Supplementary Table 6, “Methods”). Compared with Apollo samples, Chang’E-5 samples have a higher proportion of mineral contents with much less glass contents (11.5–20 wt%). The main mineral phases in the Chang’E-5 soil samples are augite, pigeonite, fayalite, forsterite, plagioclase, ilmenite, and apatite (Supplementary Table 6), among which the structure and content of the apatite grains are our focus. The apatite structure derived from the XRD pattern is P6_3_/m (Fig. 3a). Each sample was measured 20 times by XRD, and the 20 times measurements were accumulated to further avoid preferred orientation. We finally determined the apatite contents in the three soil samples were 0.1 ± 0.1 wt%, 0.7 ± 0.1 wt%, and 1.4 ± 0.1 wt%, respectively.

We further confirmed whether the apatite grains found in the Chang’E-5 returned samples contain OH. The apatite grains in the polished sections were then analyzed by Raman spectroscopy (“Methods”). Most of the apatite grains have a Raman peak that occurs near 959.3–961.7 cm^−1^ (Fig. 3b), which is caused by the fundamental vibration between P–O molecule. Influenced by the relative proportions of OH, F, and Cl, the Raman vibration mode of fluorapatite, chlorapatite, and hydroxyapatite occur at 965, 959, and 962 cm^−1^, respectively^36–38^. The Raman peak position range of the apatite grains in lunar samples suggests that a solid solution of Ca_5_(PO_4_)_3_(F, Cl, OH) is a candidate.

Based on the stoichiometric method^39,40^, the relative proportions of OH, F, and Cl in the apatite were inferred from the EPMA data. The results confirmed the presence of OH in the apatite grains, and the proportion of OH in the three endmembers of F, Cl, and OH varied from 0 to 0.38 (Supplementary Table 8). Accordingly, considering the apatite abundance in the three lunar soil samples (0.1 ± 0.1 wt%, 0.7 ± 0.1 wt%, and 1.4 ± 0.1 wt%), the corresponding hydroxyl content of these samples was estimated to be in the range of 0 ppm to 179 ± 13 ppm. This suggested that the hydroxyapatite may be unevenly distributed on lunar surface.

### Comparison between the LMS in situ rock and soils spectra

Relative to the soils, the rock is estimated to have more hydroxyl content due to its stronger 2.85 μm absorption feature. However, does this suggest that the rock does contains more hydroxyl? We first excluded the differences in hydration is compositional in origin. Comparison between the LMS in situ spectra of rock and soils shows that, except that the overall absorption depth of the rock spectrum is deeper than those of the soil spectra, they have similar spectral shapes and band centers of ~1 μm and ~2 μm, implying they are in similar composition (Supplementary Fig. 10a). Chemical analysis of CE-5 returned samples also suggested that the composition of rock fragments and soils are generally consistent^41^. Hence, we suspect that the exhibited differences in hydration between the rock and soils are attributed to their different optical properties. Comparison was also conducted on the overall absorption depth of the rock and soil spectra in similar composition from the LMS-EQM lab spectra and Apollo 11 sample lab spectra. As shown in Supplementary Fig. 10b, c, the rock spectra always have deeper ~1 μm and ~2 μm absorptions than that of soil spectra. Pieters and Noble suggested that the deeper absorption of lunar rock (fragments) relative to soil are mainly due to the difference of their surface optical properties^42^. We believe that the phenomenon also occurs on the 2.85 μm absorption band of lunar rock and soil spectra.

Although we cannot completely eliminate the case that the rock does contain more hydroxyl content than the soils, the 2.85 μm absorption depth differences between the LMS in situ rock and soil spectra are more likely due to the variations of their surface optical properties rather than the composition. In addition, it is more reasonable to choose the lunar soil spectra for comparison with orbital remote sensing data in the next section.

### Weak end of hydration features revealed by LMS soil spectra

**Fig. 4:**
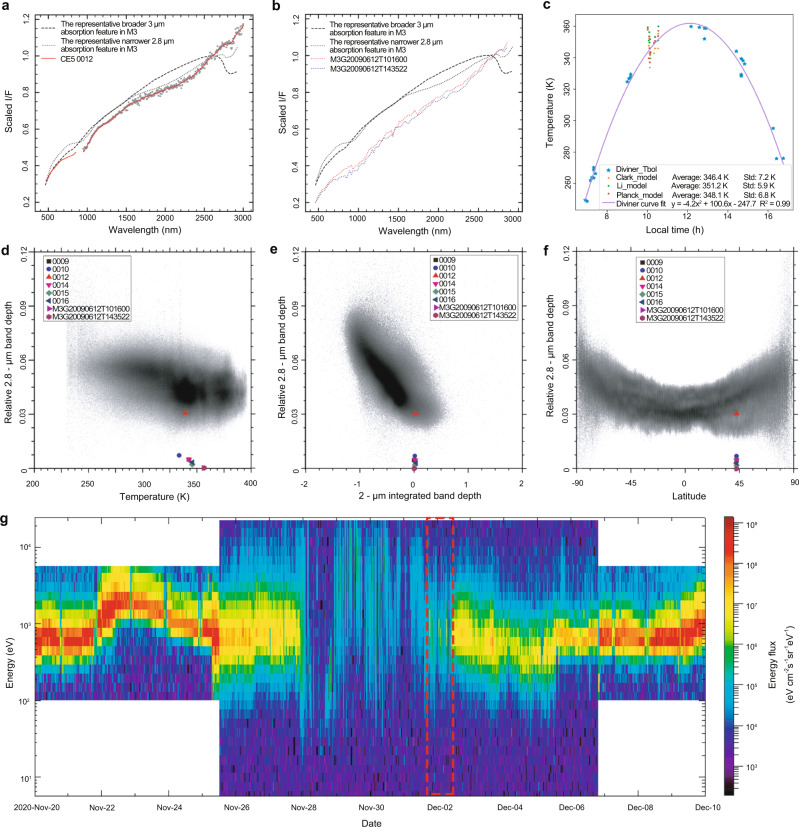
The OH absorption features comparison between the lunar mineralogical spectrometer (LMS) in situ spectra and remote sensing spectra of the Moon Mineralogy Mapper (M^3^). **a** The examples of LMS 0012 (red solid line), the representative broader 3 μm (dashed line), and 2.8 μm (dotted line) absorption features of M^3^. **b** The examples of two M^3^ spectra of Chang’E-5 landing site obtained at different times, the representative broader 3 μm (dashed line) and 2.8 μm (dotted line) absorption features of M^3^. **c** The lunar surface temperatures derived from three thermal correction models (Clark model, Li model, and Groussin model, see “Methods”) at the time of LMS in situ observations. The average temperatures derived from three models are 347.9, 351.2, and 348.1 K. The purple curve shows the variation of the lunar surface temperature with local time fitted from the Diviner data of the Chang’e-5 landing site at different lunar local times. **d**–**f** are projections of LMS ~2.85 μm band depth data to the M^3^ global ~2.81 μm band depth data varied with temperatures, 2 μm integrated band depths and latitudes (data of shaded areas, which are from Fig. 12b, Fig. 10c, and Fig. 8b of ref. ^26^). **g** ARTEMIS P1 ion energy flux spectrogram from 20 November 2020 to 10 December 2020. The area derived from the red dashed lines in the figure indicates the low energy fluxes of the received solar wind particles during LMS in situ observation period (2020.12.01–2021.12.02), when the Moon is under the protection of the Earth’s magnetosphere.

In order to specify how the Chang’E-5 sample area fits in the broader context of hydration on the lunar surface, the LMS in situ soil spectra were compared with the analysis results of M^3^ orbital remote sensing data (Fig. 4a, b, d–f). Note that the absorption centers of M^3^ are mainly located at ~2.81 μm, while the absorption centers of LMS are mainly located at ~2.85 μm. The source of the ~2.85 μm and ~2.81 μm absorption features should be consistent, both due to the OH stretching vibration. The small absorption centers difference may be related to the bond length of O–H or the stretching vibration energy^35^.

Figure 4 presents an explicit hydroxylation feature comparison with the variations of temperature, composition, and latitude. Regarding the LMS in situ soil spectra, the 2.85 μm absorptions are significantly weaker than those of M^3^ data (LMS: 0–4% vs M^3^: 3–7.5%) acquired at the same temperatures (Fig. 4d) and the same latitude (Fig. 4f). The LMS soil spectra also belong to the side representing lunar mare materials with relatively weak 2.85 μm absorptions and strong 2 μm absorption features^26^ (Fig. 4e).

We analyzed the factors that may account for the relatively weak 2.85 μm absorptions of LMS spectra. First, the LMS obtained the hyperspectral data from 10:00 to 10:30 local time, when the lunar surface temperatures range from 335 to 360 K, close to the maximum temperature at the same latitude (Fig. 4c). The lack of extensive molecular water absorption feature in the LMS spectra may thus be attributed to the fact that most of the molecular water should be evaporated under such high temperature^43^. Second, when LMS collected in situ spectra on lunar surface, the Moon is coincidentally lied within the Earth’s magnetosphere, shielded from the solar wind. The energy and flux of the solar wind reaching the lunar surface at that time were relatively low, suggesting that the contribution of solar wind hydration to the LMS in situ spectra may not be as strong as expected (Fig. 4g). Third, on a global scale, the mare spectra always show a weaker hydration absorption than the highlands spectra^26^. The LMS in situ spectra were obtained at the area filled with lunar last-stage basalts, leading to the relatively low hydroxyl contents than feldspathic materials.

The above evidences emphasized that the Chang’E-5 LMS in situ spectra may represent a weak end of the hydration features on the lunar surface. The low hydroxyl contents in lunar soil with a mean value of 28.5 ppm estimated by the LMS in situ spectra are also consistent with the no prominent hydration band observed from the telescopic full illumination spectrum^44^, and the predictions from M^3^ remote sensing data for the same latitude and time of day^12^.

### Hydroxyl sources for Chang’E-5 in situ spectra and returned samples

It is clear that the Chang’E-5 LMS spectra were acquired against a background of weak hydration. However, what the sources of the weak hydration still observed in the LMS in situ spectra? As mentioned earlier, indigenous hydroxyl, solar wind implantation, and meteorite or comet impacts are the main sources of hydroxyl on the lunar surface. Nevertheless, the hydroxyl contents in foreign materials produced by the impact processes are probably negligible. The surface regolith in Chang’E-5 landing site that is the detection target of the LMS is largely local mare material^45,46^, and the laboratory composition analysis of Chang’E-5 returned samples also suggest that the Chang’E-5 lunar soil is pure and mainly comprises in situ mare basalts^41^. Therefore, there should be little or no extraneous hydroxyl associated with impact crater or ejecta.

We then evaluated the possible contribution of the solar wind, though its role proved to be weak when the LMS collected the spectra. The content of agglutinitic glass was regarded as a measure of the solar wind contribution, because the solar wind-generated OH/H_2_O is mainly within the agglutinitic glass^23^. In an Apollo 11 soil sample (10084), ~59 wt% agglutinitic glass^47^ was measured with corresponding ~70 ppm OH/H_2_O content^23^. However, the content of agglutinitic glass in CE-5 sample is quite low (~16 wt%), only ~1/3 of that in Apollo 11 sample. Assuming that the relationship between the agglutinitic glass content and hydroxyl content is linear, the amount of solar wind-generated hydroxyl in Chang’E-5 returned samples should also be ~1/3 of that in Apollo 11 sample, which is similar to the mean hydroxyl content estimated from the LMS in situ soil spectra (28.5 ppm). This suggests that the solar wind still contributed, but indeed very weakly, to the hydroxyl contents observed in Chang’E-5 landing site.

For the soil spectra 0012 showing much higher hydroxyl content than 1/3 that of Apollo 11 sample, the excess hydroxyl content should have other source, which should be attributed to the indigenous hydroxyl. Laboratory analysis of the Chang’E-5 returned samples revealed the presence of hydroxyapatite in the sample. This strongly offers a possibility that the estimated excess hydroxyl content based on the LMS in situ spectra could be indigenous hydroxyl, and the hydroxyapatite in the soil could be one of specific hydroxyl sources.

## Methods

### The LMS and its detection

As one of the three scientific payloads of Chang’E-5 lander, the main tasks of LMS are to collect in situ spectra of the lunar surface before and after sample scooping, and to analyze the mineralogy and their distribution of the sampling area. In addition to inheriting the light dispersion method of Chang’E-3 and Chang’E-4 Visible and near-infrared imaging spectrometer (VNIS) system, the LMS extends the spectral range from 2400 to 3200 nm, and can realize lunar surface targets detection by adjusting a two-dimensional scanning motor in the pitch and yaw directions. LMS consists of a Visible and near-infrared imaging detector (VIS, 480–950 nm) and three infrared single-point detectors (NIR, 900–1450 nm; SWIR, 1400–2300 nm; MWIR, 2200–3200 nm). The main characteristics of LMS are listed in Supplementary Table 1.

Three operation modes are designed for in situ detection of LMS on lunar surface. One mode is full-bands observation (FBO), which acquires all V-NIR band images and infrared spectral data at a sampling interval of 5 nm/band for a total of 588 bands. The second operation mode is full-view scanning and multispectral observation (FVSMO), which can acquire multispectral (20 bands, Supplementary Table 2) data of multiple detection targets (180 in total, Supplementary Fig. 1) by the two-dimensional scanning motor. The last operation mode is in-flight calibration (IFC) mode, through adjusting the two-dimensional scanning motor from the lunar surface to the calibration unit, LMS can realized in situ radiometric calibration under solar illumination. The calibration unit of LMS, contains an aluminum panel and a gold panel, which are used for visible and near-infrared bands radiometric calibration and infrared bands radiometric calibration, respectively.

After Chang’E-5 landing on the surface of the Moon, drilled samples were first collected which lasted roughly for 6 h. Then, scoop sampling and LMS detection work alternately. During this period, Chang’E-5 collected scooped samples at sampling points S1, S2, and S5 (Fig. 1, where S1 was scooped 5 times, S2 was scooped 6 times, and S5 was scooped only 1 time). LMS performed 3 FVSMO, 5 IFC, and 11 FBO detections before and after sample scooping. The FBO detections include twice for the rock (detection number: 0006 and 0011) and 9 for lunar soils (twice for the S2 sampling point, detection number: 0004 and 0014; twice for the S5 sampling point, detection number: 0009 and 0015). More details are shown in Supplementary Table 3.

### Data processing of LMS

The level 2B radiance data of LMS were used in this study, which underwent dark-current subtraction, scattering-background correction, flat field, instrument temperatures correction, radiometric calibration (including in situ radiometric calibration), and geometric calibration. We first calculated the radiance factor (RADF) data of the lunar surface using the solar irradiance^48^ as the following:

1 $${{{{{{\rm{RADF}}}}}}}\left(i,e,g\right)=\frac{{{{{{\rm{I}}}}}}}{{{{{{\rm{F}}}}}}}\left(i,e,g\right)=\frac{\pi I}{J/{d}^{2}}$$

Here, *I* is the radiance, *J* is the solar irradiance of the top of the Earth’s atmosphere, and *d* is the solar-Moon distance which is about 0.988 AU during the working time of LMS on lunar surface. Since the VIS detector and the NIR detector share the same field of view and the SWIR and MWIR also share the same field of view, the reflectance spectra of all the 256 × 256 image pixels in the VIS bands were averaged to obtain one spectrum (480–950 nm). This averaged spectrum was then connected to the point spectrum of the NIR, SWIR, and MWIR bands. However, the spectral jitter caused by different responses of the four detectors at the connection bands should be corrected. Considering the stability of the NIR spectral data, we took NIR data as the standard and adjusted all the other three detectors’ data to the NIR data to obtain one continuous spectrum. The overlapping bands between NIR and SWIR are from 1400 to 1450 nm, and the overlapping bands between SWIR and MWIR are from 2200 to 2300 nm. Because NIR and SWIR have the same spectral trends at their corresponding overlapping bands, the adjustment factor for the NIR/SWIR was obtained by averaging the ratios of NIR data to SWIR data for their overlapping bands. Similar process was conducted to obtain the adjustment factor for the SWIR/MWIR. For the connection between VIS and NIR bands, the following method was conducted to obtain the adjustment factor. First, we found that the data after 950 nm of the NIR channels and the data before 860 nm of the VIS channels are reliable, but the data in the range of 860–950 nm are unreliable and should be removed (Supplementary Fig. 2a). Then, the VIS (480–855 nm) and NIR (950–1100 nm) spectral data were fitted separately with the quadratic polynomial, and the band values from 860 to 950 nm for both VIS and NIR were extended following their corresponding quadratic polynomial fitted trends. After that, the ratios of NIR to VIS were calculated between 860 nm to 950 nm at 1 nm intervals (Supplementary Fig. 2b). Finally, the minimum ratio from 860 to 950 nm was selected as the adjustment factor for the VIS/NIR channel, and the whole continuous reflectance spectra of the rock and soils are shown in Supplementary Fig. 2c, d.

### Thermal correction

The thermal emission after 2000 nm of the lunar surface cannot be ignored. If thermal effect were not considered, the calculated reflectance based on the lunar surface radiation will deviate from the true values. The absorption features of hydrated minerals at longer wavelengths will be also strongly affected or obscured by the thermal emission of lunar surface^49^, especially when the LMS works at high temperatures (for example, >400 K near the Moon’s equator at noon). Unfortunately, it was just close to noon when LMS worked on the lunar surface, and the thermal emission of lunar surface is extremely intense caused by the high temperature. Therefore, it is necessary to conduct thermal correction for LMS data.

Three thermal correction models were applied in our work, including Clark’s model^32^, Li and Milliken’s model^33^, and Groussin’s model^34^. We first repeated and validated the three thermal correction models using the M^3^ and HRIIR data (Supplementary Fig. 3). Then, the three models were used to remove the thermal component of the LMS FBO data (Supplementary Fig. 4).

First, based on the linear relationship of reflectance data in the range of 1500–3200 nm shown by Clark et al., the reflectance of LMS at 1700, 2350, 2280, and 2600 nm were iteratively used to estimate the thermal corrected reflectance of LMS at 3100 nm. The reflectance data with and without thermal correction at 3100 nm were both put into Eq. (2) in each iteration, and the temperature can be derived after multiple iterations when the temperature variations are <2 K.

In Eq. (2), *R* is the reflectance before thermal correction, *R*_0_ is the true reflectance after thermal correction, *J* is the solar irradiance, *B*_(*T*)_ is the Planck function at temperature *T*, and *e* is the emissivity of the lunar surface. According to the Kirchhoff’s law, *e* = 1−*R*_0_.

2 $$R={R}_{0}+\frac{\pi e{B}_{(T)}}{J}$$

Second, the model of Li and Milliken (2016) based on the empirical law derived from Apollo lunar samples was used, which is shown in Eq. (3).

3 $${R}_{2540}=1.124\times {R}_{1550}^{0.8793}$$

Here, *R*_2540_ and *R*_1550_ are the reflectance values at 2540 and 1550 nm, respectively. Through Eq. (3), the thermal corrected reflectance at 2540 nm can be obtained. Then, this value and the value before thermal correction were both put into Eq. (2).

At last, the model of Groussin et al. was used to directly fit Planck function for constraining the thermal component (also called Planck model). The thermal contribution was removed from each spectrum by independently fitting and subtracting a blackbody function using data beyond 2.5 μm, which also provides an estimate of the temperature for each spectra. Emissivity was also assumed to equal one at all wavelengths. Since the actual emissivity must be <1, this simplification results in an underestimate of the temperature by ~5 K^10^.

The derived temperatures of LMS observation after thermal correction on the lunar surface are between 335 and 360 K, and they fit well with the trend predicted by the Diviner data of the Chang’e-5 landing site at different lunar local times (Fig. 4c). But the average temperature of the lunar surface derived from Li’s model is 3 K higher than the other two models’ results.

As shown in Supplementary Fig. 4, the reflectance corrected by the three different thermal correction methods all include significant downturns at bands over 3100 nm, which are inconsistent with previous work (e.g., Deep Impact and OSIRIS-REx) and lab data. However, the downturns may not be caused by the thermal correction methods. After further examination of the data (as shown in Supplementary Fig. 5), we found that the rising trend after 3100 nm of the LMS in situ spectra before thermal correction gradually slowed down, while the trend of model fit data with reflected and thermal components gradually increased. Their discrepancy increased when making thermal correction on LMS spectra, leading to the significant downturns at bands over 3100 nm. The upward trend of the LMS reflectance after 3100 nm slowed down significantly mainly because this band is at the end of the detection range of the LMS instrument and the spectral response was reduced. The same phenomenon was found in the ground test data of the LMS prior to its launch into space. Relative to the standard comparison spectrometers (ASD and DP102F), the LMS spectral data in the 3100–3200 nm range showed significantly slower upward trends due to the reduced spectral response. In view of this situation, we only analyzed and discussed the LMS spectral data ≤3100 nm. Since the hydration features discussed in our work occur before 3100 nm, using the spectra ≤3100 nm will not affect the analysis of hydroxyl and its content. We thus only discuss the spectra with the wavelength shorter than 3100 nm in this study.

### Photometric correction

According to the working mode of the FVSMO, LMS obtained multispectral data of 180 detection targets in the field of view (Supplementary Fig. 1). The phase angles of these multispectral data cover from 21° to 94°. The Lommel-Seeliger model was adopted for photometric correction in this study, and the data were corrected to the standard viewing geometry (incidence angle = 30°, emittance angle = 0°, and phase angle = 30°) using the following equations.

4 $$\frac{I}{F}=\frac{{\mu }_{0}}{{\mu }_{0}+\mu }f(g)$$

5 $$f\left(g\right)={a}_{3}{g}^{3}+{a}_{2}{g}^{2}+{a}_{1}g+{a}_{0}$$

6 $$\frac{I}{F}(30^\circ ,0^\circ ,30^\circ )=\frac{I}{F}(i,e,g)\frac{{\cos }30^\circ /({\cos }30^\circ +{\cos }0^\circ )}{{\cos }i/({{{{{{\rm{cos}}}}}}i}+{\cos }e)}\frac{f(30^\circ )}{f(g)}$$

First, reflectance calculation and thermal correction were also conducted to the multispectral data of LMS. Second, those image data which contain rocks, shadows, and saturated pixels and the data of poor SNR were excluded. A total of 146 multispectral detection targets remained after this process. Then, a three-order polynomial were used to fit the phase function of Eq. (5) to obtain the fitting coefficients for all the 20 bands of the multispectral data (Supplementary Table 4). Finally, the multispectral reflectance data were corrected to the standard viewing geometry according to Eq. (6). In addition, the same bands of the 11 FBO data can be directly corrected using the above functions, and the coefficients of other bands could be interpolated by these 11 bands data. The full-range spectra of the 11 FBO data are shown in Supplementary Fig. 6d.

From the Supplementary Fig. 6d, we can clearly see typical ~1 μm and ~2 μm absorption features in the LMS in situ spectra, as well as the hydration absorption feature at ~2.85 μm. We also found an unexpected absorption feature at ~2550 nm in the first three LMS in situ spectra (0004, 0006, and 0008), and the other eight spectral data collected subsequently did not have such absorption feature. Further comparative analysis revealed that the overall albedos of these three spectral data (0.08–0.12) were also significantly higher than those of the other eight spectra (0.02–0.10). We suspected that these three spectral data may have been interfered by other scattered light during data collection. For this reason, we examined the operation of the robotic arm at the moment of spectral measurement, and confirmed the movement of the robotic arm in the spectral detection region. The shadow left by the robotic arm can be seen in Fig. 1b. We believed that the albedo anomalies and the 2.5 μm anomalous absorptions in these three spectral data may be due to the effect of scattered light produced by the robotic arm movement. Therefore, we did not discuss these three spectral data in the main text.

### Analysis of the ~3 μm absorption features

We first analyzed whether the absorption features of LMS near 2.85, 2.95, and 3.05 μm are from the lunar surface in this study. First, it is known that two calibration panels are carried by LMS (gold panel and aluminum panel) which are used for in situ radiometric calibration. LMS performed three times of full-bands radiometric calibration during its work on the lunar surface. The three reflectance curves of the two calibration panels are shown in Supplementary Fig. 7. It can be seen from Supplementary Fig. 7 that the 2.85, 2.95, and 3.05 μm absorption features are not observed for the calibration panels. This suggests that the absorptions detected by LMS originates from lunar surface rather than the calibration panels. In addition, the first two calibrations were carried out earlier, and the panels did not exhibit obvious thermal emission. However, in the third calibration, the panels have been exposed to the sun for about 16 h and the thermal effect is obvious indicating a much higher temperature of the lunar surface (According to the result of thermal correction, the temperature of lunar surface at that time should be 335–360 K). Second, the fuel carried by Chang’E-5 lander is methylhydrazine and dinitrogen tetroxide. The plume eruption during the landing process will produce water and carbon dioxide, and the chemical reaction formula can be written as 4CH_3_NHNH_2_+5N_2_O_4_ = 9N_2_+4CO_2_+12H_2_O. The water produced by the plume may be adsorbed on the lunar surface. However, under a typical temperature ~360 K of Moon surface, most of the adsorbed water will volatilize within half an hour and the content of adsorbed water will be close to 0 ppm^50^. The residual adsorbed water is difficult to be detected by spectroscopy method. Since the first spectrum of LMS was acquired 8.5 h after landing, nearly all the plume water should be vaporized and the spectra of LMS should not be affected by the plume water. Therefore, we believed that these three absorption features of LMS spectra are from lunar surface rather than from the lander plume.

The sources about the 2.95 and 3.05 μm absorptions seen in the LMS in situ spectra are complex and may be related to the absorptions of H_2_O. The 2.95 μm absorption can be attributed to asymmetric and symmetric stretching modes of the H_2_O molecule or to OH bonds, depending on the bonding cations or bond energies^10^. The 3.05 μm absorption may be related to H–O–H bending vibration absorption of water molecules^35^, which could be derived from the non-structural, OH and H_2_O in nominally-anhydrous minerals, from the OH and H_2_O in glasses or H_2_O in fluid inclusions, or from the physisorbed and chemisorbed H_2_O on the lunar surface^35^. However, these two absorptions are too weak to be identified and quantified. Therefore, only the 2.85 μm hydroxyl absorption was discussed in this work.

### Hydroxyl content estimation

We Estimated the hydroxyl content of LMS spectral data using the method of Li and Milliken (2017). First, LMS data were corrected for the thermal and photometric effects, and the Single Scattering Albedo (SSA) was calculated based on the Hapke theory (Eq. 7, Supplementary Fig. 8a–h). Parameters used here can be referred to Li and Li^51^. After that, the SSA were smoothed by the Savitzky-Golay algorithm and the continuum were removed by Convex Hull method (Supplementary Fig. 8i). Then, the ESPAT values at the wavelength of 2850 nm was calculated (Eq. 8). Finally, the hydroxyl content was derived by the empirical formula (Eq. 9) which obtained from step-wise heating experiments in the laboratory. The hydroxyl contents of the detection targets are shown in Supplementary Table 5.

Since the hydroxyl contents calculated by three correction methods are similar (Supplementary Table 5), and the hydroxyl content based on the Clark thermal correction method is in the middle of the three models, we only analyzed and discussed the hydroxyl content calculated by the Clark’s method in the main text.

7 $${{{{{{\rm{RADF}}}}}}}=\frac{\omega }{4}\frac{{\mu }_{0}}{{\mu }_{0}+\mu }\{\left[1+B\left(g\right)\right]P\left(g\right)+H\left(\omega ,\mu \right)H\left({\mu }_{0},\omega \right)-1\}$$

8 $${{{{{{\rm{ESPAT}}}}}}}=\frac{1-\omega }{\omega }$$

9 $${{{{{{\rm{H}}}}}}}_{2}{{{{{\rm{O}}}}}} \% =0.59\times {{{{{{\rm{ESPAT}}}}}}}$$

### Lunar powder samples and polished section samples preparation

Three powder samples and three rock-fragment polished section samples were prepared for laboratory analysis. The powder sample1 is from Chang’E-5 lunar soil CE5C0800YJFM001, the powder sample2 and sample3 are both from Chang’E-5 lunar soil CE5C0100YJFM002. The processes for preparing the three powder samples are as the following: First, the lunar soils (CE5C0800YJFM001 and CE5C0100YJFM002) placed in the quartz bottles were scooped out and put into three grooves which are with a diameter of 20 mm and a depth of 5 mm. Then, large soil grains visible to the naked eyes were picked out by a tweezer, and the powder samples were stirred adequately and scraped to flat surfaces by the quartz glass sheets. Each powder sample is weighted about 100 mg. After that, the powder samples were used for the analysis of XRD and the EQM of LMS. The three rock-fragment polished sections were processed through the following processes: rock fragments selection, sticking to the glass sheet, glue injection, vacuum infiltration, grinding and polishing, coating, etc. After these processes, the three polished sections were analyzed by SEM, EDS, EPMA, and Raman Spectrometer. The sample numbers of the three polished sections are CE5C0000YJYX042GP, CE5C0000YJYX063GP, CE5C0000YJYX03501GP.

### Spectral analysis of Lunar powder and rock samples using the LMS-EQM

The three powder samples and a CE-5 rock-fragment sample (sample ID: CE5C0000YJYX105) were analyzed by the Engineering Qualification Module (EQM) of LMS (LMS-EQM). In order to save samples, we must first adjust the light path and the field of view of LMS-EQM to satisfy the need for a 2 cm diameter powder sample. The dust cover and calibration panels of the LMS-EQM are required to be removed, and then the viewing direction of the LMS-EQM should be adjusted to the horizontal direction. After that, an off-axis parabolic mirror is needed to install on the LMS-EQM ensuring the view of the LMS-EQM vertical to the sample surface. At last, an additional external optics is also required to force the observation field of LMS-EQM to focus on a small scale to meet the requirement of 2 cm diameter. The spectra of the three powder samples ranging from 480 to 3200 nm obtained by the LMS-EQM are shown in Supplementary Fig. 9a, b. According to Clark et al., under the laboratory temperature (~300 K), the thermal components have little influence on the LMS-EQM lab reflectance spectra in the range of 2.8–3 μm (reflectance change <0.002), so the thermal effect can be ignored. In addition, Because the intensity of the laboratory xenon light is significantly weaker than the sunlight and can result in an obvious low SNR of LMS in the range of 480–900 nm, only the spectral data between 900 and 3100 nm was adopted in this study. After continuum removal, the spectra of the lunar powder samples in the laboratory also exhibit the 2.85 μm absorption indicating the existence of hydroxyl. However, the absorption features observed in the laboratory are much broader than that for in situ spectra, which may be partly related to the influence of water vapor in the light path of the LMS-EQM.

Several absorptions occur in the LMS-EQM lab measured spectra of CE-5 samples around 1900 nm, 2200–2300 nm, and 2600–2700 nm, which have not been identified and discussed in this study. Among them, 2200–2300 nm is the connection bands between the LMS-EQM’s third (Short Wave Infrared (SWIR): 1400–2300 nm) and fourth (Mid Wave Infrared (MWIR): 2200–3200 nm) channels. The spectral responses in this range could be instable and anomalies could exist. Thereby, the features appearing in this range cannot be accurately identified and were not discussed in this study. Although 1900 nm and 2600–2700 nm absorptions are not observed in the LMS in situ data, but they can be found in the LMS-EQM lab measured spectra of CE-5 returned samples. After further examination, we believe that these two absorptions may reflect the influence of water vapor in the atmospheric environment during the laboratory measurement of LMS-EQM. We compared the spectral data of the CE-5 returned samples using LMS-EQM (spectral measurement range 950–3200 nm) and standard comparison instrument ASD (spectral measurement range 480–2500 nm) under the same atmospheric environment condition (Supplementary Fig. 9a), it is found that the LMS_EQM lab spectra have absorption characteristics around 1900 nm and 2600–2700 nm, while these characteristics do not exist in the ASD lab spectra. We inferred the discrepancy is due to the different measurement conditions of the two instruments. In the process of measurement, the ASD probe is very close to the surface of the sample and the calibration panel, and the full spectrum was acquired in a very short time (<1 s), which was little affected by the water vapor environment in the detection light path. However, the design of LMS-EQM keeps its probe away from the measured sample surface and the calibration panel (~7 cm away), and LMS-EQM would take ~10 min to obtain a full spectrum. Therefore, atmospheric water vapor in the detection light path easily produces the absorption characteristics at 1900 nm and 2600–2700 nm. The above analysis indicates that the absorption characteristics around 1900 nm and 2600–2700 nm could not exist or be identified in the LMS in situ spectra. Therefore, they are not discussed in this study

Comparison between the LMS-EQM lab spectra of CE-5 returned samples and Apollo 11 lunar soil lab spectra shows that they both have a broad OH/H_2_O absorption characteristic at 2.7–3.2 μm (Supplementary Fig. 9b, c). However, as shown in Supplementary Fig. 9b, the spectral slope of Apollo 11 spectra is larger than that of LMS-EQM spectra towards the longer wavelengths (>1500 nm, spectral redder), indicating that Apollo soil is more mature.

We compared the absorption features between the lunar rock and soil for both LMS in situ spectra and LMS-EQM laboratory spectra. Besides, the spectra of Apollo 11 rock-fragment (sample ID: LS-JBA-001-P2) and soil (LSCC 10084) samples were also compared. Both the LMS in situ and LMS-EQM lab measurements show that lunar rock and soil have similar spectral shapes and absorption centers, but the rock has deeper ~1 and ~2 μm absorptions than that of soil (Supplementary Fig. 10a, b). This is also consistent with what is observed by the spectral measurements of Apollo samples (Supplementary Fig. 10c).

In addition, three spectra of hydroxyl-containing apatite were found in RELAB spectral library (Supplementary Fig. 11), and their absorptions at ~2.85 μm are consistent with LMS in situ spectra. this result imply that hydroxyl contained apatite could be contributable to the ~2.85 μm hydration absorption features of LMS in situ spectra.

### SEM and EDS analysis of lunar polished section samples

Back-scattered electron (BSE) images were collected using a Carl Zeiss SUPRA-55 Field Emission Scanning Electron Microscopy (FESEM) at the National Astronomical Observatories, Chinese Academy of Sciences in Beijing. The probe current was 300 pA at an accelerating voltage of 15 kV. To search for apatite grains, the mineral phases were identified with FESEM equipped with qualitative energy-dispersive spectroscopic (EDS) analysis. As shown in Fig. 2.

### XRD analysis of lunar powder samples

The identification and quantification of the mineral phases of powder samples are analyzed by Bruker D8 Advanced X-ray diffraction instrument and the Rietveld full-pattern fitting method at the National Astronomical Observatories, Chinese Academy of Sciences in Beijing. The homogeneous and fine powder samples are critical for obtaining quality diffraction patterns. Large particles that are visible to the naked eyes were picked out and the powder samples were fully stirred (at least 1 h) to make homogenous mixing and to avoid preferred orientation. After that, the samples were scraped to a flat surface before XRD measurement. The measurement conditions set for the XRD are as the following: the 2*θ* angles range from 5° to 90°, the increment is set to be 0.015°, and the time for each step is 0.5 s, and the whole pattern of each sample will cost ~1 h. The XRD measurement will repeat 20 times and the data of the 20 measurements will be accumulated to further avoid preferred orientation. The three powder samples’ XRD diffraction patterns and the Rietveld full-pattern fitting results by Jade software are shown in Supplementary Fig. 12, the mineral phases identified and the contents calculated are listed in Supplementary Table 6. The mineral phases identified and involved in the full-pattern Rietveld fitting included augite, pigeonite, plagioclase, forsterite, fayalite, ilmenite, quartz, and apatite. The standard diffraction patterns of each mineral phase are from the International Center for Diffraction Data (ICDD), and the corresponding card number for each mineral phase is listed in Supplementary Table 7. The full-pattern fitting errors (weighted-profile R-factor, Rwp) are 6.06%, 5.04%, and 5.77%, respectively.

### Raman spectral analysis of lunar polished section samples

The apatite grains in the three polished sections are analyzed by Raman spectroscopy. A HR Evolution laser Raman spectrometer (HORIBA company) was used for imaging and measurement at the National Astronomical Observatories, Chinese Academy of Sciences in Beijing. A total of 11 points of 5 apatite grains in the three polished sections were analyzed (Supplementary Table 8), Part of the locations of analyzed apatite particles are shown in Fig. 2c. In this measurement, a 532 nm Nd: YAG laser is focused on the sample, and a ×100 objective is used, which can generate an ~1-μm diameter laser spot on the samples. The wavelength range was set between 50 and 1400 cm^−1^, and each spectrum acquisition time was set as 3 s. The number of acquisitions was set as 2. After each Raman spectrum obtained, the baseline correction and peak finding operation were performed. And part of the results was shown in Fig. 3b.

### EMPA analysis of lunar polished section samples

Quantitative chemical analyses of apatite grains were conducted using a JEOL JXS-8100 electron-probe micro-analyzer (EMPA) in wavelength-dispersive spectroscopy (WDS) at the institute of geology and geophysics, Chinese Academy of Science. An accelerating voltage of 15 kV and a 2.14 × 10^−8^ A beam current were used for the analysis of apatite and the standards. The 12 standards were selected including natural silicate minerals, phosphate minerals, and metallic oxide minerals. A total of 11 points of 5 apatite grains in the three polished sections were analyzed, and OH were found in 6 points. The OH is calculated by stoichiometry based on 13 anions^52^, and the range of OH ratios calculated in the three endmembers (F, Cl, OH) is 0–0.38. All the data has been corrected for ZAF (atomic number, X-ray absorption, fluorescence) effect^53^, and the details of the 11 points are shown in Supplementary Table 8.

## Supplementary information

Supplementary Information

## Data Availability

The 11 FBO hyper-spectra data obtained by LMS in situ detection and the three Chang’E-5 lunar soil samples spectra obtained by the EQM of the LMS in the laboratory are provided in Source data files. Besides, the XRD pattern of the three Chang’E-5 lunar soil samples and the Raman spectra of the apatite grains in the polished sections are also provided in Source data files. The standard diffraction patterns of each mineral phase are from the International Center for Diffraction Data (ICDD, https://www.icdd.com/). Datasets generated or analyzed during this study are available from the corresponding author upon reasonable request. Source data are provided with this paper.

## Source data

Source Data
