# Supplementary material for: Evidence of water on the lunar surface from Chang’E-5 in-situ spectra and returned samples
Source: Nat Commun. 2022 Jun 14;13:3119. doi: 10.1038/s41467-022-30807-5 (PMC9198042; doi:10.1038/s41467-022-30807-5)
Supplement: Supplementary file 1 — Supplementary Information [file 41467_2022_30807_MOESM1_ESM.pdf]

SUPPLEMENTARY INFORMATION

Evidence of Water on the Lunar Surface from Chang'E-5 In-situ Spectra and Returned Samples

Jianjun Liu[1,2#], Bin Liu[1#], Xin Ren[1], Chunlai Li[1,2*], Rong Shu[3*], Lin Guo[1,2], Songzheng Yu[1,2], Qin Zhou[1], Dawei Liu[1], Xingguo Zeng[1], Xingye Gao[1], Guangliang Zhang[1], Wei Yan[1], Hongbo Zhang[1,2], Lihui Jia[4], Shifeng Jin[5], Chunhua Xu[5], Xiangjin Deng[6], Jianfeng Xie[7], Jianfeng Yang[8], Changning Huang[9], Wei Zuo[1,2], Yan Su[1,2], Weibin Wen[1], Ziyuan Ouyang[1,10]

1 Key Laboratory of Lunar and Deep Space Exploration, National Astronomical Observatories, Chinese Academy of Sciences, Beijing, 100101, China
2 University of Chinese Academy of Sciences, Beijing 100049, China
3 Key Laboratory of Space Active Opto-electronics Technology, Shanghai Institute of Technical Physics, Chinese Academy of Sciences, Shanghai 200083, China
4 State Key Laboratory of Lithospheric Evolution, Institute of Geology and Geophysics, Chinese Academy of Sciences, Beijing 100029, China
5 Beijing National Laboratory for Condensed Matter Physics, Institute of Physics, Chinese Academy of Sciences, Beijing 100190, China
6 Beijing Institute of Spacecraft System Engineering, Beijing 100094, China
7 Beijing Aerospace Control Center, Beijing 100094, China
8 Xi'an Institute of Optics and Precision Mechanics, Chinese Academy of Sciences, Xi'an, 710119, China
9 Beijing Institute of Space Mechanics Electricity, China Academy of Space Technology, Beijing 100076, China
10 Institute of Geochemistry, Chinese Academy of Sciences, Guiyang, 550081, China
# These authors contributed equally to this work.
** Corresponding authors: licl@nao.cas.cn, shurong@mail.sitp.ac.cn*

Supplementary Figures

Supplementary Fig.1
Supplementary Fig.2
Supplementary Fig.3
Supplementary Fig.4
Supplementary Fig.5
Supplementary Fig.6
Supplementary Fig.7
Supplementary Fig.8
Supplementary Fig.9
Supplementary Fig.10
Supplementary Fig.11
Supplementary Fig.12

Supplementary Tables

Supplementary Table 1
Supplementary Table 2
Supplementary Table 3
Supplementary Table 4
Supplementary Table 5
Supplementary Table 6
Supplementary Table 7
Supplementary Table 8

Supplementary Figures

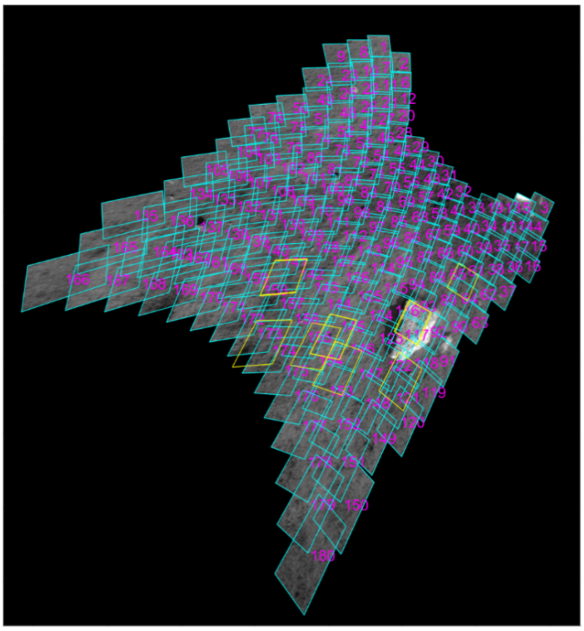

Supplementary Figure 1. The detection of the Lunar Mineralogical Spectrometer (LMS) Full-View Scanning and Multispectral Observation (FVSMO) and its detection numbers (1~180)

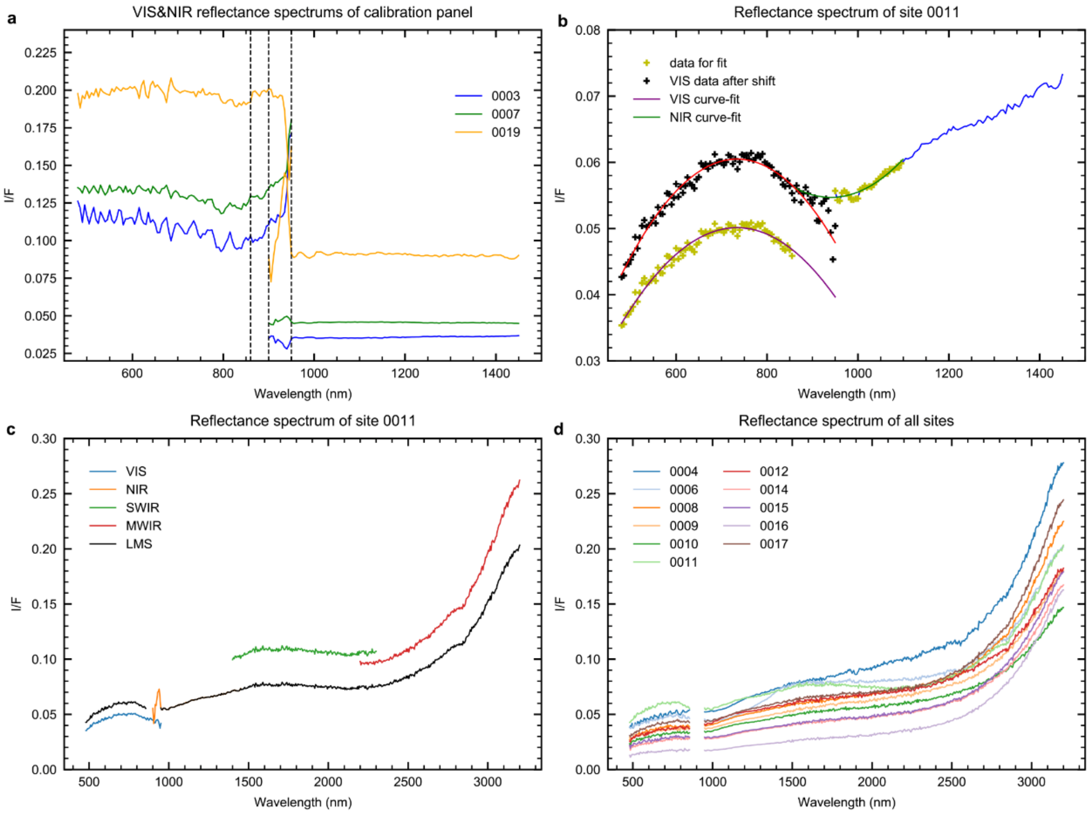

Supplementary Figure 2. Schematic diagram of reflectance spectra processing. a, the three reflectance spectra of the calibration panels (spectral range 480-1400nm) show the obvious spectral jitter in the range of 860-950nm. The three black vertical dash lines are located at 860nm, 900nm and 950nm; **b,** schematic of obtaining the Visible and Near-Infrared (VIS/NIR) adjustment factor (take 0011 data for example); **c,** schematic of connecting four channels data to obtain one continuous spectra (take 0011 data for example); **d,** all the 11 Full-Bands Observation (FBO) spectra after data processing.

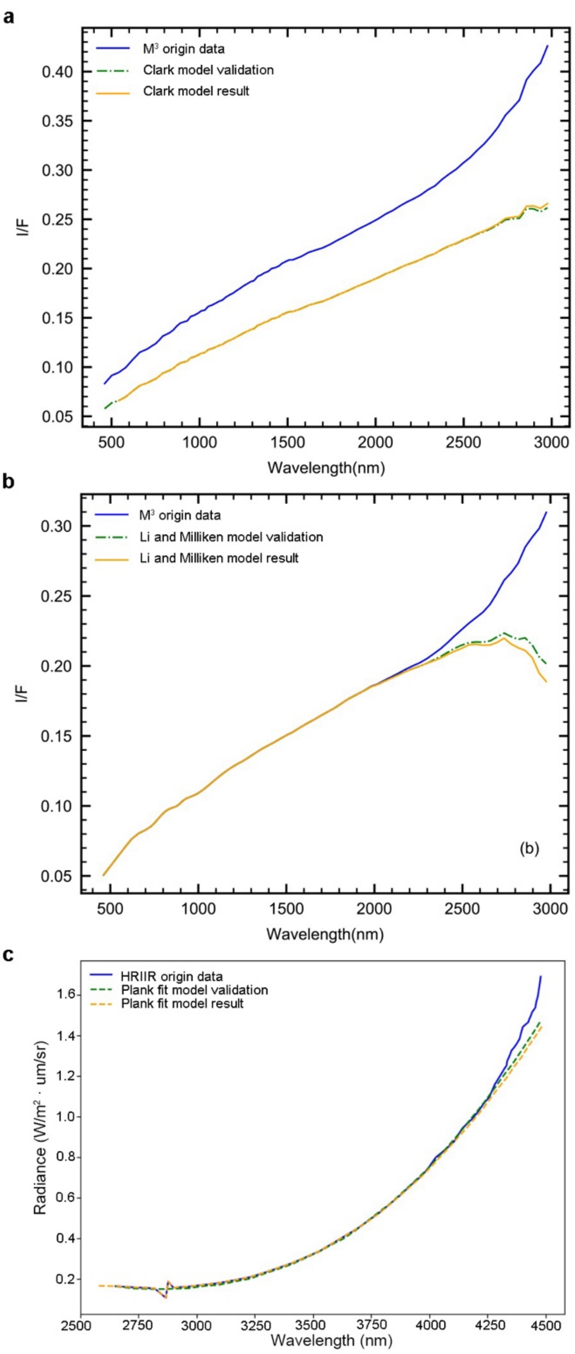

Supplementary Figure 3. The validation of the three thermal correction methods using the Moon Mineralogy Mapper (M^3) and High-Resolution Instrument-infrared spectrometer (HRIIR) data. The blue solid lines show the data before thermal correction, the yellow dashed lines demonstrate the data after thermal correction (**a** and **b**) or thermal model fit data (**c**), and the green dashed lines exhibit validated thermal correction results. **a,** the validation of the thermal correction using the Clark et al. (2011) model; **b,** the validation of the thermal correction using Li and Milliken (2016) model. **c,** the validation of thermal model fit using Groussin's model (Planck model). When repeating the Li and Milliken (2016) thermal correction model, the correction result has a deviation of ~6% near 3000nm. Since we do not know the accurate position and topographic information of the M^3 data used in the literature, this deviation may be caused by the inaccurate photometric correction.

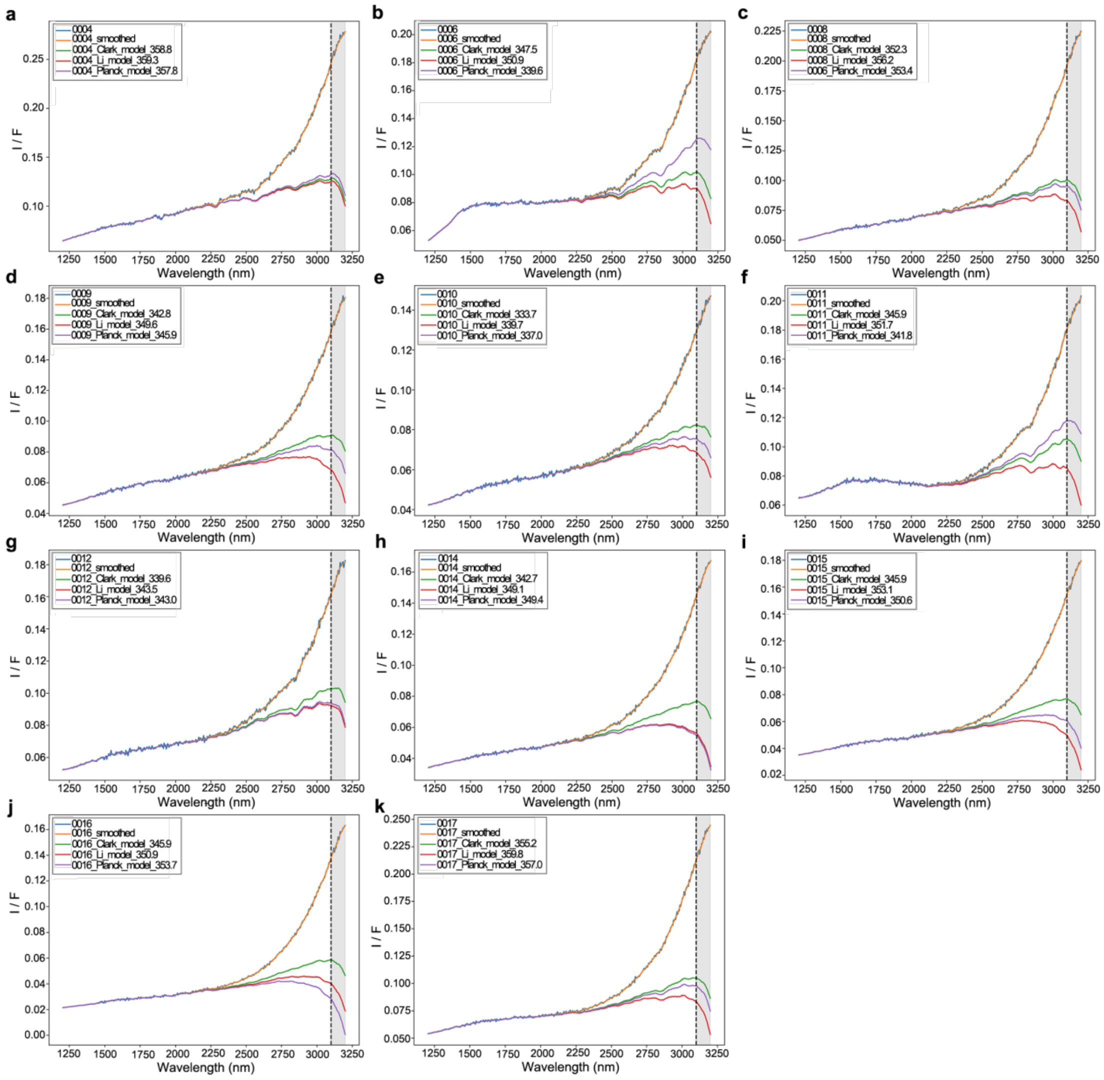

Supplementary Figure 4. The 11 unsmoothed (blue lines), smoothed (yellow lines) and thermally corrected the Lunar Mineralogical Spectrometer (LMS) in-situ spectra using the three thermal correction models (Clark model: green lines; Li model: red lines; Groussin model: purple lines). A significant downturn existed in the thermally corrected data longward of 3100nm. Almost all the spectra exhibit 2.85μm absorption features excepted 0009 and 0015, which show very weak absorption features.

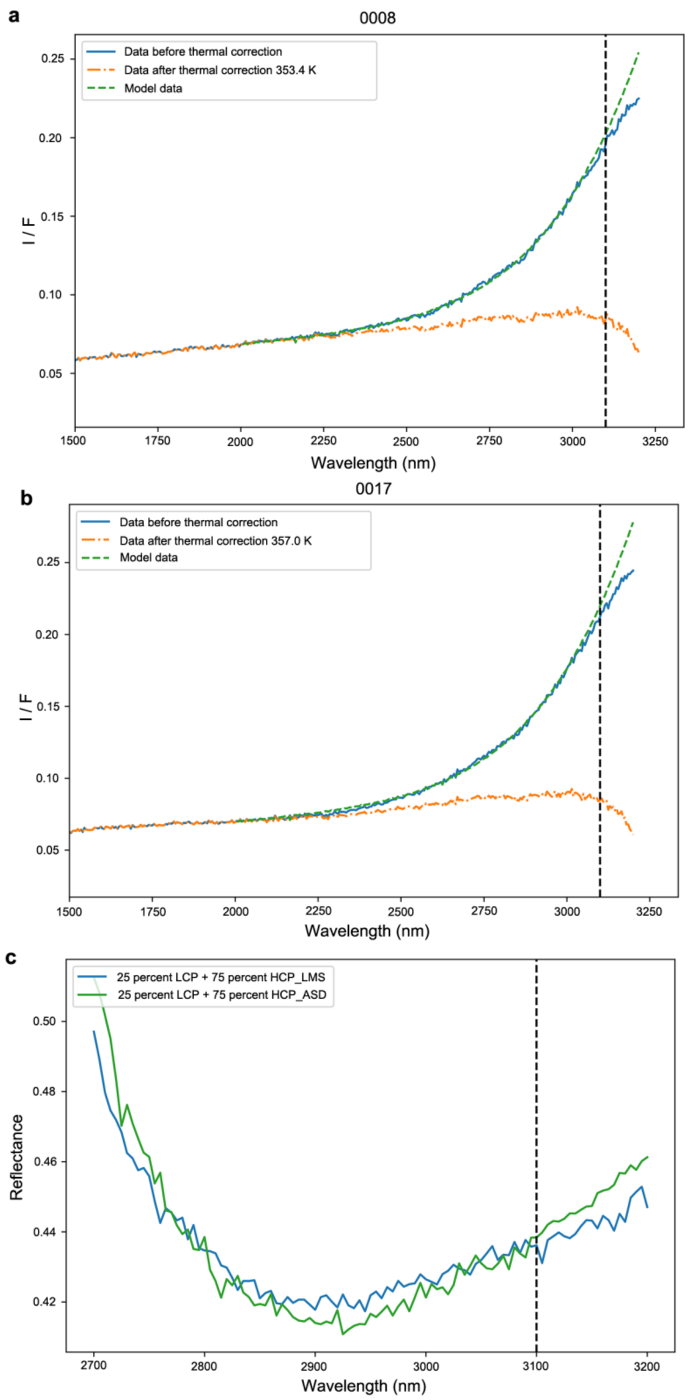

Supplementary Figure 5. The slowdown of the upward trend after 3100 nm in the Lunar Mineralogical Spectrometer (LMS) in-situ spectra and ground test data. a and **b** show that the rising trend after 3100 nm of the LMS in-situ spectra 0008 and 0017 before thermal correction gradually slowed down, while the trend of model fit data with reflected and thermal components gradually increased. Their discrepancy increased when conducting thermal correction on LMS spectra, which leads to the significant downturns at bands over 3100 nm. **c,** The same phenomenon was found in the ground test data of the LMS prior to its launch into space.

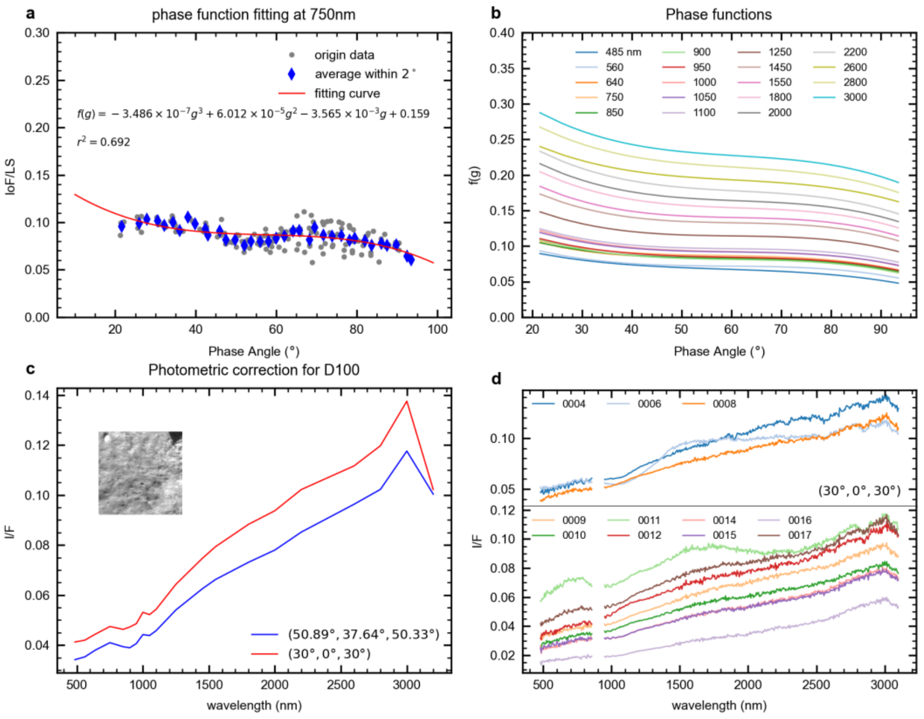

Supplementary Figure 6. Schematic of photometric correction. a, Phase function fitting (band 750 nm); **b,** Phase function fitting results of all the 20 bands of multi-spectral data; **c,** Photometric correction of multi-spectral data (take target 100 for example with its image in the up-left corner); **d,** The 11 Full-Bands Observation (FBO) data after photometric correction (the data in the 860-945 nm range are discarded), the spectra of 0004, 0006 and 0008 are listed in the upper panel, which show unexpected absorption features at 2300nm, 2550nm.

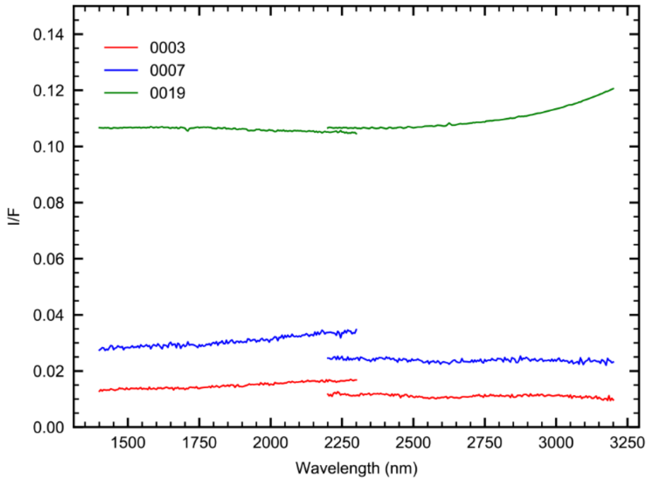

Supplementary Figure 7. The three in-situ reflectance spectra of the calibration panels. The first two calibrations were carried out earlier, and the panels did not exhibit obvious thermal emission. But in the third calibration, the panels have been exposed to the sun for about 16 hours and the thermal effect is obvious.

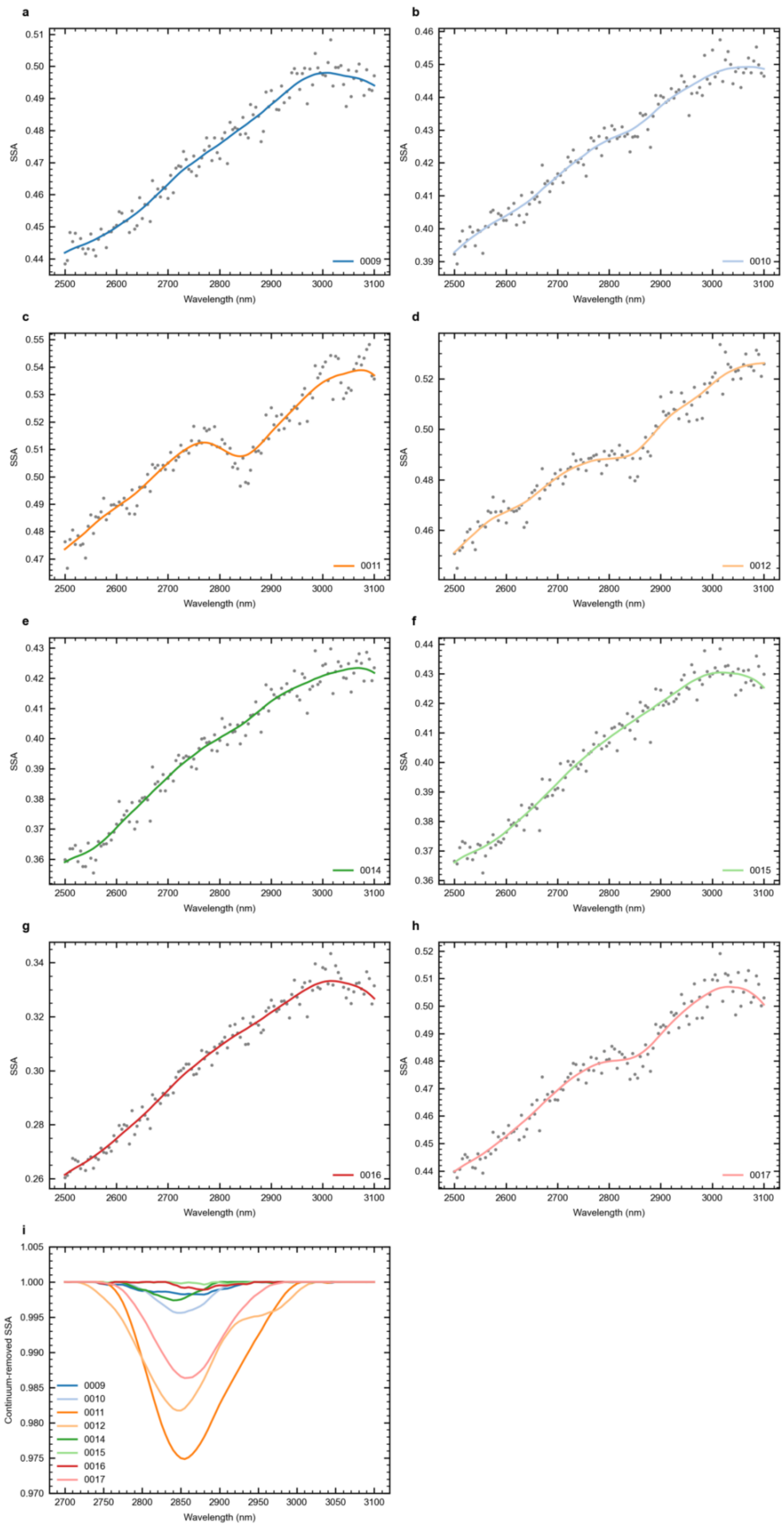

Supplementary Figure 8. The derived Lunar Mineralogical Spectrometer (LMS) in-situ Single Scattering Albedo (SSA) spectra (2500-3100nm). The dots in sub images **a-h** are original unsmoothed SSA spectra, and the colored solid lines in sub images **a-h** are smoothed SSA spectra, and the 8 smoothed SSA spectra after continuum removal are shown in sub image i.

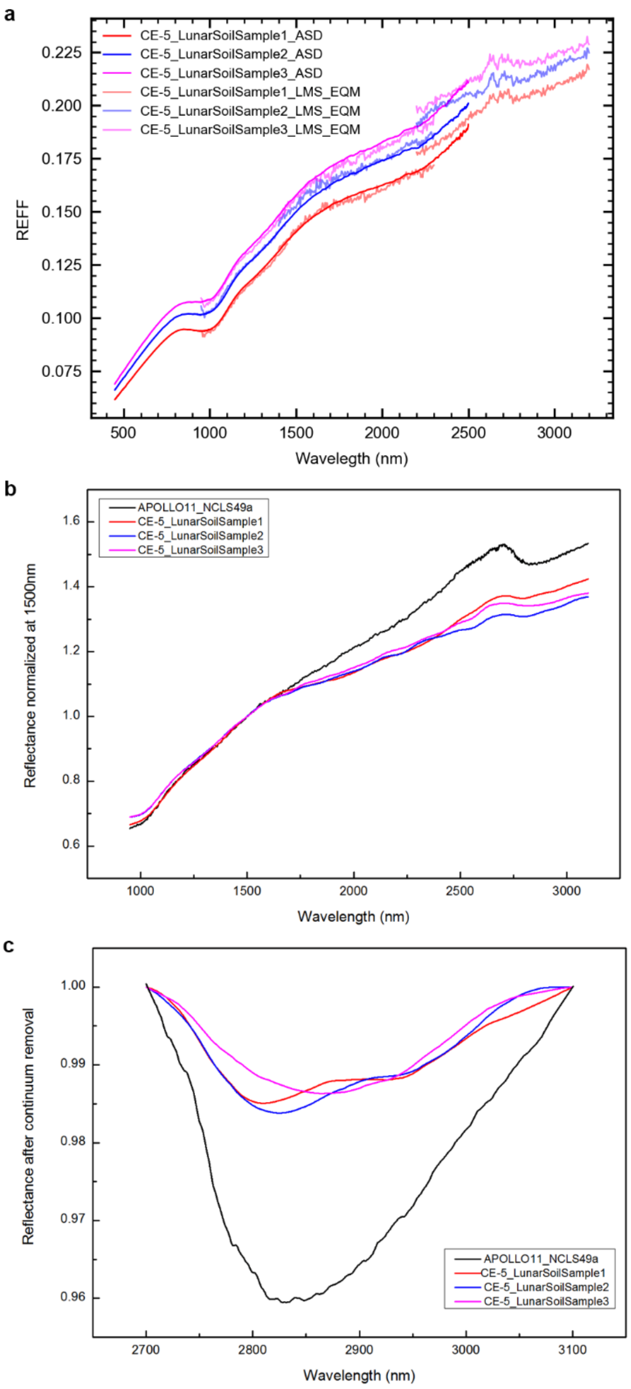

Supplementary Figure 9. Comparison among the Engineering Qualification Module of Lunar Mineralogical Spectrometer (LMS_EQM) spectra of Chang'E-5 (CE-5) lunar soil samples, the Analytical Spectral Devices (ASD) spectra of CE-5 lunar soil samples and the lab spectra of APOLLO 11 mare soil samples. a, Comparison between the LMS_EQM spectra and ASD spectra of CE-5 lunar soil samples; **b,** Comparison between the spectra of CE-5 lunar soil samples and APOLLO 11 mare soil samples; **c,** comparison between the continuum removed spectra (2700-3200nm) of CE-5 lunar soil samples and APOLLO 11 mare soil samples.

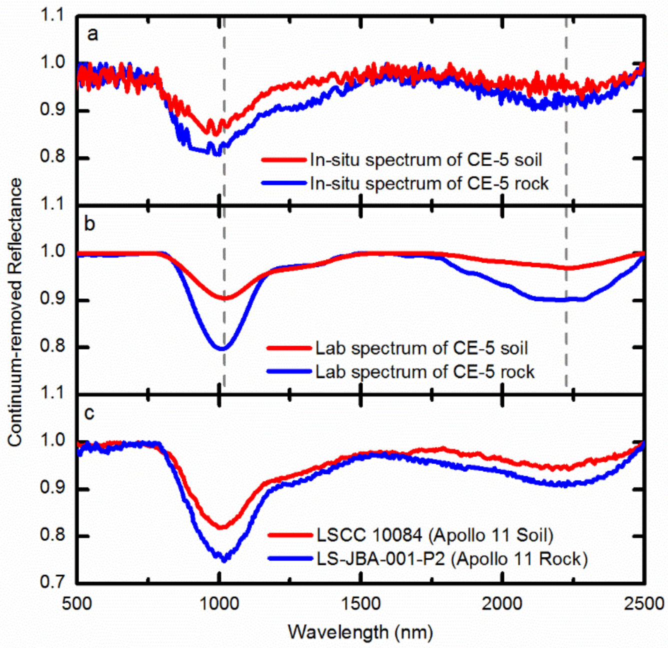

Supplementary Figure 10. Absorption characteristics comparison between the rock and soil for Chang'E-5 and APOLLO missions. a, absorptions comparison between the rock and soil for Chang'E-5 in-situ spectra; **b,** absorptions comparison between the Chang'E-5 rock (fragments) and soil samples for Chang'E-5 lab spectra; **c,** absorptions comparison between the rock and soil spectra of Apollo 11samples.

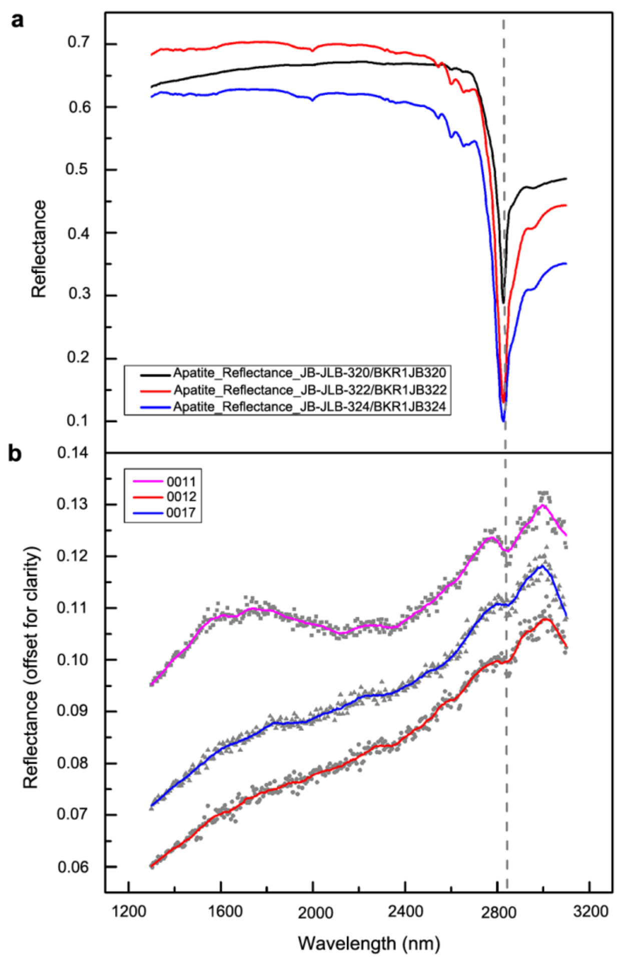

Supplementary Figure 11. The ~2.85μm absorption features comparison between the hydroxyapatites' spectra and the Lunar Mineralogical Spectrometer (LMS) in-situ spectra (the spectral data of the hydroxyapatites are come from Reflectance Experiment Laboratory (RELAB), http://www.planetary.brown.edu/relabdocs/relab.htm)**.**

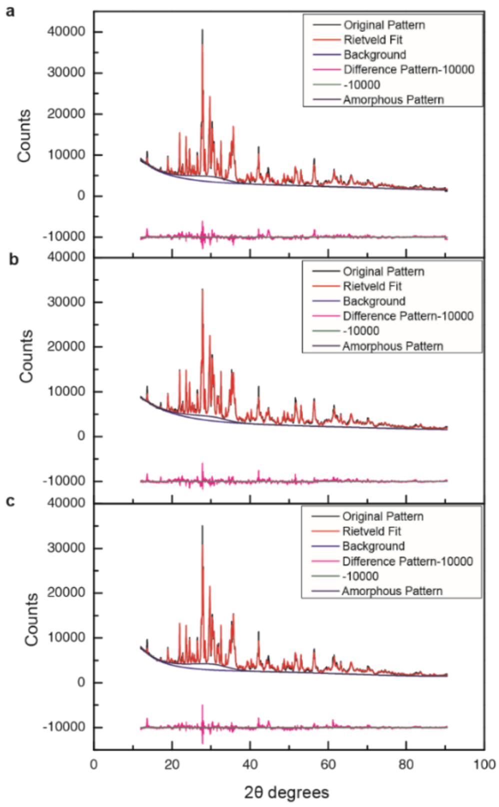

Supplementary Figure 12. X-Ray diffraction spectra and Rietveld full-pattern fitting results of the three lunar soil samples. a is Lunar Soil Sample1 sourced from the Chang'E-5 lunar soil CE5C0800YJFM001; **b, c** are Lunar Soil Sample2 and Lunar Soil Sample3, which soured from the Chang'E-5 lunar soil CE5C0100YJFM002.

Supplementary Tables

Supplementary Table 1. Main characteristics of the LMS

Parameters	Results			
	VIS	NIR	SWIR	MWIR
Spectral range（nm）	480~950	900~1450	1400~2300	2200~3200
Spectral resolution（nm）	3~12	3~12	8~24	10~25
Field of view（°）	4.17×4.17			
S/N（dB）	Peak⩾55, minimum 34			
Modular transfer function	⩾0.14			
Detect distance（m）	2～5			

Supplementary Table 2. Bands selection of Full-View Scanning and Multi-spectral Observation (FVSMO) mode

Channel	Band Wavelength（nm）
VIS	485,560,640,750,850,900
NIR	950,1000,1050,1100,1250,1450
SWIR	1550,1800,2000,2200
MWIR	2600,2800,3000,3200

Supplementary Table 3. LMS operations at the lunar surface

Detection Number	Starting time (UTC)	Stopping time (UTC)	Detection mode and target information
0001	2020-12-01T21:08:12	2020-12-01T21:15:46	FBO (Self-check)
0002	2020-12-01T21:20:13	2020-12-01T22:16:03	FVSMO (VIS/NIR bands), 1~180 targets in Supplementary Fig.1
0002	2020-12-01T22:08:23	2020-12-01T22:16:02	FVSMO (SWIR bands), 1~180 targets in Supplementary Fig.1
0003	2020-12-01T22:18:39	2020-12-01T22:26:13	1st IFC (Full-bands), calibration panels
0004	2020-12-01T23:53:03	2020-12-02T00:00:46	FBO, S2 sampling site, lunar soil, the spectra were collected before scooping, the surface is not disturbed by scooping, and the position is the same as 0014
0005	2020-12-02T00:42:24	2020-12-02T00:52:08	FVSMO (MWIR bands), 1~180 targets in Supplementary Fig.1
0006	2020-12-02T01:11:08	2020-12-02T01:18:45	1st FBO of the big rock, and the position is the same as 0011
0007	2020-12-02T01:33:11	2020-12-02T01:40:52	2nd IFC (Full-bands), calibration panels
0008	2020-12-02T02:02:49	2020-12-02T02:10:31	FBO, after sampling at S1, Lunar soil, the surface is not disturbed by scooping
0009	2020-12-02T02:20:15	2020-12-02T02:27:57	FBO, S5 sampling site, lunar soil, the spectra were collected before scooping, the surface is not disturbed by scooping, and the position is the same as 0015
0010	2020-12-02T02:52:14	2020-12-02T02:59:57	FBO, Lunar soil, the surface is not disturbed by scooping
0011	2020-12-02T03:12:15	2020-12-02T03:19:58	2nd FBO of the big rock, and the position is the same as 0006
0012	2020-12-02T03:41:41	2020-12-02T03:49:24	FBO, lunar soil, the spectra were collected before scooping, the surface is not disturbed by scooping
0013	2020-12-02T04:00:11	2020-12-02T04:02:12	3rd IFC (only VIS/NIR bands), calibration panels
0014	2020-12-02T09:02:27	2020-12-02T09:10:09	FBO, S2 sampling site, lunar soil, the spectra were collected after six scoop sampling times, the surface is disturbed by scooping, and the position is the same as 0004
0015	2020-12-02T10:43:01	2020-12-02T10:50:29	FBO, S5 sampling site, lunar soil, the spectra were collected after one scoop sampling, the surface is disturbed by scooping, and the position is the same as 0009
0016	2020-12-02T15:05:28	2020-12-02T15:12:56	FBO, Lunar soil, and the position is close to S2 sampling site, the surface is disturbed by scooping
0017	2020-12-02T15:20:29	2020-12-02T15:27:57	FBO, Lunar soil, part of the rock in the view, the surface is disturbed by scooping.
0018	2020-12-02T15:34:04	2020-12-02T15:35:40	4th IFC (only VIS/NIR bands), calibration panels
0019	2020-12-02T16:56:43	2020-12-02T17:04:10	5th IFC (Full-bands), calibration panels

Supplementary Table 4. Fitting coefficients of the phase functions

Wavelength (nm)	$a_3(10^{-7})$	$a_2(10^{-5})$	$a_1(10^{-3})$	a_0	R^2
485	-2.641	4.621	-0.003	0.134	0.796
560	-3.628	6.432	-0.004	0.150	0.748
640	-4.128	7.147	-0.004	0.166	0.731
750	-3.486	6.012	-0.004	0.159	0.692
850	-3.381	5.971	-0.004	0.159	0.738
900	-4.105	7.409	-0.005	0.177	0.745
950	-3.807	6.838	-0.004	0.173	0.735
1000	-4.301	7.775	-0.005	0.193	0.728
1050	-4.137	7.448	-0.005	0.187	0.702
1100	-4.342	7.819	-0.005	0.195	0.691
1250	-5.223	9.450	-0.006	0.234	0.677
1450	-6.002	10.911	-0.007	0.273	0.683
1550	-6.204	11.359	-0.007	0.290	0.687
1800	-6.285	11.647	-0.007	0.317	0.724
2000	-6.293	11.654	-0.007	0.329	0.714
2200	-6.387	11.824	-0.008	0.350	0.719
2600	-5.254	9.605	-0.006	0.335	0.647
2800	-6.292	11.550	-0.008	0.382	0.661
3000	-6.925	12.711	-0.008	0.412	0.636
3200	-1.386	1.365	0.000	0.186	0.241

Supplementary Table 5. The hydroxyl content of in-situ spectral data (unit: ppm)

Detection number	Spectral obtaining time (lunar local time)	Derived temperatures on the lunar surface (K)			Calculated water contents (ppm)		
		Using the model by Clark et al (2011)[32]	Using the model by Li and Milliken (2016)[33]	Using the model by Groussin (2007)[34]	Using the model by Clark et al (2011)[32]	Using the model by Li and Milliken (2016)[33]	Using the model by Groussin (2007)[34]
0009	10:07	344	350	346	11	2	5
0010	10:08	335	340	337	26	18	23
0011	10:08	348	352	342	152	138	186
0012	10:10	341	343	343	110	93	96
0014	10:20	344	349	349	15	6	6
0015	10:24	347	353	351	2	1	2
0016	10:32	347	351	354	7	2	1
0017	10:33	357	360	357	82	68	85

Supplementary Table 6. The identification and quantification of mineral phases in the three powder samples

Minerals	Samples' name and minerals' contents（wt. %）		
	Lunar Soil Sample1	Lunar Soil Sample2	Lunar Soil Sample3
Plagioclase	29.1	31.8	29.3
Augite	28.8	35.0	28.8
Pigeonite	13.3	9.1	10.9
Forsterite	2.0	1.5	1.4
Fayalite	3.5	4.9	4.0
Ilmenite	4.6	4.3	4.6
Apatite	0.1	1.4	0.7
Quartz	0.4	0.4	0.3
Glass	18.2	11.6	20.0

Supplementary Table 7. Minerals used in the Rietveld full-pattern fitting and the corresponding Powder Diffraction Files (PDF) of ICDD

	Mineral	PDF numbers of ICDD
1.	Plagioclase	PDF04-011-6816
2.	Augite	PDF98-000-0102
3.	Pigeonite	PDF01-076-2962
4.	Forsterite	PDF01-085-1207
5.	Fayalite	PDF04-008-8540
6.	Ilmenite	PDF04-007-2813
7.	Apatite	PDF01-080-7126
8.	Quartz	PDF98-000-0369

Supplementary Table 8. EMPA and Raman measurements of apatite grains in the three polished sections. The OH is calculated by stoichiometry. A total of 11 points of 5 apatite grains were analyzed by EMPA and Raman spectra. (take #063-3-1 for example, #063-3-1 means that first measurement point of EPMA or Raman on the third apatite grain in the polished section numbered CE5C0000YJYX063GP).

	**CE5C0000YJYX063GP**					**CE5C0000YJYX042GP**			**CE5C0000YJYX03501GP**		
	**#063-1-1**	**#063-3-1**	**#063-3-2**	**#063-3-4**	**#063-3-5**	**#042-1-1**	**#042-1-2**	**#042-1-3**	**#03501-2-2**	**#03501-3-1**	**#03501-3-2**
**wt.%**											
P_2O_5	41.80	41.135	40.474	41.703	41.603	41.045	37.974	41.108	40.214	39.213	39.26
SiO_2	1.93	1.761	1.213	1.19	1.343	1.48	6.915	1.993	2.494	3.548	4.955
SO_3	0.00	0.00	0.00	0.00	0.00	0.00	0.00	0.00	0.00	0.00	0.00
MgO	0.08	0.22	0.186	0.249	0.285	0.006	0.017	0.023	0.055	0	0
CaO	51.36	50.974	51.842	53.243	51.864	52.059	47.794	50.321	51.121	51.624	50.494
FeO	0.98	0.988	1.199	1.277	1.916	1.786	1.918	1.92	1.801	2.492	2.383
MnO	0.04	0.037	0.107	0.05	0.07	0.051	0.07	0	0.066	0.06	0.066
Na_2O	0.16	0.13	0.1	0.094	0.079	0.029	0.079	0.009	0.053	0	0
Cr_2O_3	0.00	0.013	0	0	0	0	0.027	0.012	0	0.019	0.003
Al_2O_3	0.26	0.77	0.39	0.354	0.126	0.155	0.825	0.21	0.198	0.272	0.496
K_2O	0.13	0.038	0.015	0.034	0	0.136	0.553	0.159	0.059	0.097	0.22
NiO	0.01	0	0.008	0.018	0.049	0.003	0	0	0	0	0
TiO_2	0.01	0.038	0.03	0.076	0.04	0.557	0.132	0.48	0.034	0.226	0.131
SrO	0.00	0.00	0.00	0.00	0.00	0.00	0.00	0.00	0.00	0.00	0.00
Cl	1.40	1.539	1.205	0.985	1.385	0.489	0.265	0.355	0.193	0.467	0.391
F	2.43	2.76	3.877	3.825	2.562	2.858	3.017	3.547	4.115	3.613	2.011
F_2=-O	-1.02	-1.16	-1.63	-1.61	-1.08	-1.20	-1.27	-1.49	-1.73	-1.52	-0.85
Cl_2=-O	-0.32	-0.35	-0.27	-0.22	-0.31	-0.11	-0.06	-0.08	-0.04	-0.11	-0.09
Total	99.40	98.89	98.74	101.27	100.02	99.52	98.42	98.56	98.63	100.00	100.12

	CE5C0000YJYX063GP					CE5C0000YJYX042GP			CE5C0000YJYX03501GP		
	#063-1-1	#063-3-1	#063-3-2	#063-3-4	#063-3-5	#042-1-1	#042-1-2	#042-1-3	#03501-2-2	#03501-3-1	#03501-3-2
**Stoichiometry based on 13 anions**											
P	3.19	3.18	3.21	3.21	3.19	3.15	2.90	3.19	3.15	3.03	2.94
SiO_2	0.17	0.16	0.11	0.11	0.12	0.13	0.62	0.18	0.23	0.32	0.44
SO_3	0.00	0.00	0.00	0.00	0.00	0.00	0.00	0.00	0.00	0.00	0.00
MgO	0.01	0.03	0.03	0.03	0.04	0.00	0.00	0.00	0.01	0.00	0.00
CaO	4.97	4.99	5.21	5.18	5.03	5.05	4.62	4.94	5.07	5.05	4.79
FeO#	0.07	0.08	0.09	0.10	0.15	0.14	0.14	0.15	0.14	0.19	0.18
MnO	0.00	0.00	0.01	0.00	0.01	0.00	0.01	0.00	0.01	0.00	0.00
Na_2O	0.03	0.02	0.02	0.02	0.01	0.01	0.01	0.00	0.01	0.00	0.00
Cr_2O_3	0.00	0.00	0.00	0.00	0.00	0.00	0.00	0.00	0.00	0.00	0.00
Al_2O_3	0.00	0.00	0.00	0.00	0.00	0.00	0.01	0.00	0.00	0.00	0.00
K2O	0.01	0.00	0.00	0.00	0.00	0.02	0.06	0.02	0.01	0.01	0.02
NiO	0.00	0.00	0.00	0.00	0.00	0.00	0.00	0.00	0.00	0.00	0.00
TiO_2	0.00	0.00	0.00	0.01	0.00	0.06	0.01	0.05	0.00	0.02	0.01
SrO	0.00	0.00	0.00	0.00	0.00	0.00	0.00	0.00	0.00	0.00	0.00
Cl	0.21	0.24	0.19	0.15	0.21	0.08	0.04	0.06	0.03	0.07	0.06
F	0.69	0.80	1.15	1.10	0.73	0.82	0.86	1.03	1.21	1.04	0.56
OH	0.10	-0.04	-0.34	-0.25	0.06	0.10	0.10	-0.09	-0.23	-0.11	0.38
